# Oxadiazole/Pyridine-Based Ligands: A Structural Tuning for Enhancing G-Quadruplex Binding

**DOI:** 10.3390/molecules23092162

**Published:** 2018-08-28

**Authors:** Filippo Doria, Valentina Pirota, Michele Petenzi, Marie-Paule Teulade-Fichou, Daniela Verga, Mauro Freccero

**Affiliations:** 1 Dipartimento di Chimica, Università di Pavia, 27100 Pavia, Italy; filippo.doria@unipv.it (F.D.); valentina.pirota01@universitadipavia.it (V.P.); michele.petenzi@gmail.com (M.P.); 2 Institut Curie, PSL Research University, CNRS UMR9187, INSERM U1196, F-91405 Orsay, France; mp.teulade-fichou@curie.fr; 3 Paris Sud University, Paris-Saclay University, CNRS UMR9187, INSERM U1196, F-91405 Orsay, France

**Keywords:** G-quadruplex, oxadiazole/pyridine polyheteroaryls, G4-ligands, FRET-melting, G4-FID, circular dichroism

## Abstract

Non-macrocyclic heteroaryls represent a valuable class of ligands for nucleic acid recognition. In this regard, non-macrocyclic pyridyl polyoxazoles and polyoxadiazoles were recently identified as selective G-quadruplex stabilizing compounds with high cytotoxicity and promising anticancer activity. Herein, we describe the synthesis of a new family of heteroaryls containing oxadiazole and pyridine moieties targeting DNA G-quadruplexes. To perform a structure–activity analysis identifying determinants of activity and selectivity, we followed a convergent synthetic pathway to modulate the nature and number of the heterocycles (1,3-oxazole vs. 1,2,4-oxadiazole and pyridine vs. benzene). Each ligand was evaluated towards secondary nucleic acid structures, which have been chosen as a prototype to mimic cancer-associated G-quadruplex structures (e.g., the human telomeric sequence, c-myc and c-kit promoters). Interestingly, heptapyridyl-oxadiazole compounds showed preferential binding towards the telomeric sequence (22AG) in competitive conditions vs. duplex DNA. In addition, G4-FID assays suggest a different binding mode from the classical stacking on the external G-quartet. Additionally, CD titrations in the presence of the two most promising compounds for affinity, TOxAzaPy and TOxAzaPhen, display a structural transition of 22AG in K-rich buffer. This investigation suggests that the pyridyl-oxadiazole motif is a promising recognition element for G-quadruplexes, combining seven heteroaryls in a single binding unit.

## 1. Introduction

G-quadruplex DNAs (G4s) are tetra-helical structures arising in nucleic acid sequences containing repeats of three or four adjacent guanines, self-assembling into square co-planar arrays (G-tetrads), through Hoogsteen hydrogen bonding [1]. Several G-tetrads, upon K^+^ coordination [2,3] can stack on each other to form a G4, with the interconnecting sequences extruded as single-strand loops of various length [4,5]. It is well documented that these structures exist in a conformational equilibrium with the single stranded DNA domains transiently generated during key biological processes, such as telomere maintenance, recombination, replication, transcription and epigenetic regulation [6], together with genome instability [7,8]. Therefore, G4s have been the object of intense study with the aim of defining their potential as regulatory elements and/or therapeutic targets. Thus, G4s are considered druggable targets that offer the possibility to control these fundamental processes in cells [9]. Organic compounds capable of recognizing and stabilizing G4 structures have attracted great attention as selective probes and anticancer agents since they are assumed to act selectively at genomic G-rich loci such as telomeres and oncogene promoters [10], which have higher propensity to fold into G-quadruplex structures than other putative quadruplex sequences (PQSs) within the human genome. Dual selectivity towards G4 versus *ds* DNA and different G4 architectures is a key aspect for developing efficient G4 DNA binding drugs and diagnostic tools. Nowadays, we are far from being able to identify G4 ligands binding selectively a given G4 structure. Nevertheless, small molecules able to induce down-regulation of transcription of specific oncogenes, due to the targeting of G4 structures localized within gene promoters, are reported in the literature. In this context, the downregulation of c-kit by benzo[a]phenoxazines [11], c-myc by the ellipticin analog GQC-05 [12], and HSP90 by phenyl bis-oxazole derivatives [13] are paradigmatic examples.

**Figure 1:**
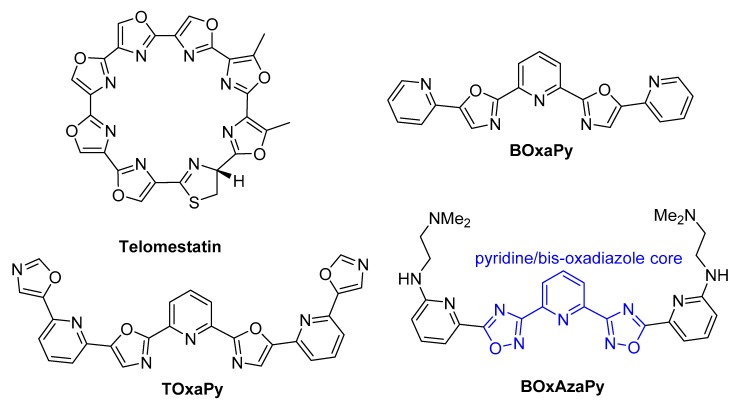
Structure of Telomestain, together with non-macrocyclic and flexible oligoaromatics previously evaluated as G4 ligands [21,22].

**Scheme 1:**
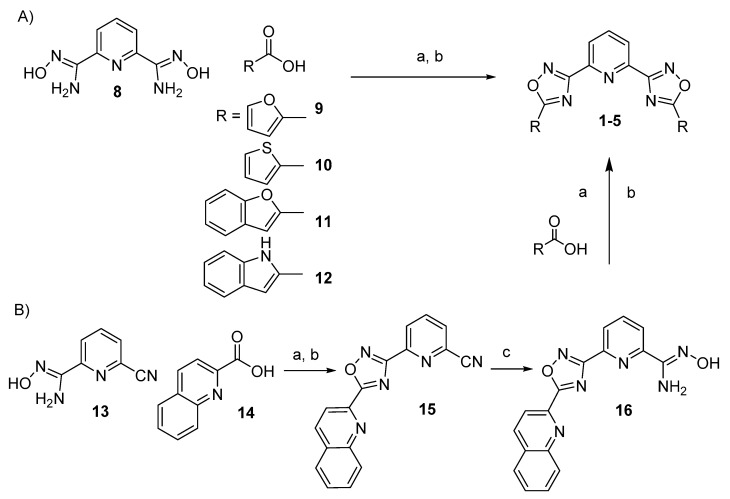
Synthetic protocol developed for the preparation of the penta-heterocyclic compounds: (**A**) one-step synthetic pathway followed for the preparation of compounds **1**–**4**; and (**B**) multi-step synthetic pathway for the preparation of compound **5** (a) 1,1’-carbonyldiimidazole (CDI), dry DMF, 16 h, r.t.; (b) 1,1’-carbonyldiimidazole (CDI), dry DMF, 6 h, 150 °C; and (c) NH_2_OH/Na_2_CO_3_, H_2_O, 1 h, r.t.).

A massive synthetic effort has been focused on the development of potent and selective G4 ligands, the main class of compounds being represented by extended planar aromatic compounds able to interact by π-stacking with the external quartets. Often, to increase binding affinity and water solubility, these ligands are functionalized with cationic moieties or with lateral chains that can be protonated in physiological conditions [14,15,16,17]. However, at present, a limited number of binders simulates the peculiar characteristics of the natural macrocycle telomestatin [18] (Figure 1), which exhibits excellent binding properties and remarkable antitumor activity. Its unique molecular features inspired the design and synthesis of a large number of both macrocyclic [19,20] and non-macrocyclic ligands (Scheme 1) [13,21,22,23,24,25]. Among them, the non-macrocyclic binders with high rotational flexibility are rather underrepresented as a class of G-quadruplex ligands. However, the high rotational flexibility of these oligoaryls should increase the number of possible binding modes occurring between the ligand and the G4 structures, from classical π-stacking to grove binding or hybrid interactions (Figure 1). The initial structure–activity investigation reported for these polyaromatic binders focused on the study of the effect of ligand length on target affinity, suggested that the neutral symmetric (BOxaPy and TOxaPy, Figure 1) and asymmetric pyridyl-oxazole motif, particularly when consisting of a minimum of five alternating units and cationic appendages, serves as a potent binding element for G-quadruplexes recognition [21,23,24].

**Figure 2:**
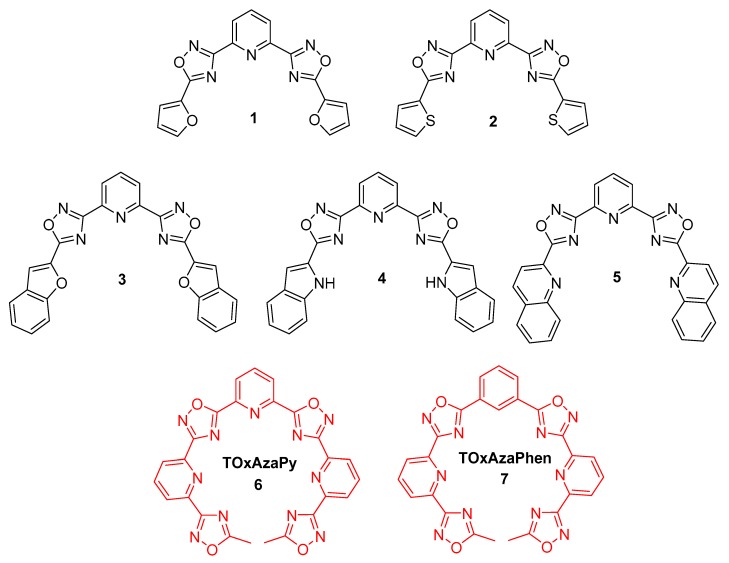
Structures of the newly synthesized non-macrocyclic penta-hetero-aromatics **1**–**5** and hepta-hetero-aromatics TOxAzaPy (**6**) and TOxAzaPhen (**7**).

Although these penta-aromatic compounds are modest stabilizers of pre-folded G-quadruplex structures, they could be further optimized by exploiting a synthetic strategy that allows the functionalization of the structures with amino/cationic side-chains (BOxAzaPy, Figure 1) [22]. These promising results prompted us to develop a pool of ligands characterized by conformational flexibility (**1**–**7**, Figure 2), allowing adaptability to the G-quadruplex DNA target. In particular, starting from the “lead” pyridyl-oxadiazole central core, we focused our attention on the number of aromatic rings and the introduction of different heteroaryl units, with 1,2,4-oxadiazole being the ever present structural moiety. Herein, we describe the synthesis of a small library of these oligo-hetero-aromatics also outlining the preliminary biophysical data: FRET melting and G4-FID assays collected in the presence of prototype G4s chosen to mimic cancer-associated G4s. The effect produced by these structural changes on G4 selectivity and stabilization has been evaluated and the most potent ligands were then further investigated by circular dichroism (CD) titration to appraise their ability to induce conformational changes.

## 2. Results and Discussion

### 2.1. Design and Synthesis of the Polyheteroaryl Oxadiazole/Pyridine-Ligands

To implement the potency and selectivity of the BOxAzaPy G4 ligand and assess the effect of the nature of the aromatic rings composing the ligand on G4 binding, we introduced different heterocycles directly bound to the central pyridine/bis-oxadiazole core (Figure 2), performing in a single step the synthesis of both oxadiazoles (Pathway A, Scheme 1). The synthesis is a one-pot process constituted by an initial *O*-acylation followed by a cycloaromatization. The condensation between pyridine-2,6-bis-amidoxime **8** and a small set of carboxylic acids (**9**–**12** and **14**, Scheme 1) occurred in the presence of 1,1′-carbonyldiimidazole (CDI) as a coupling reagent. The subsequent cycloaromatization was achieved without isolation of the resulting intermediate O-acylbenzamidoxime, generating the final BOxAzaPys **1**–**4** in low yields (Table 1). However, this one-pot protocol yielded the quinolinium oxadiazole **5** in very low yields (<5%), as a sludge residue was recovered, probably due to the oligomerization of **8**. Therefore, the synthesis of **5** was more effectively optimized by a stepwise process, introducing the oxadiazole rings one at the time (Pathway B, Scheme 1). The (*Z*)-6-cyano-*N*′-hydroxypicolinimidamide **13** was initially *O*-acylated by the carboxylic acid **14**, and the resulting intermediate cycloaromatized to the cyano-oxadiazole **15** (Scheme 1). The cyano group was subsequently converted into amidoxime **16** in the presence of hydroxylamine, which yielded the final product **5** after *O*-acylation and cycloaromatization, as described above.

**Scheme 2:**
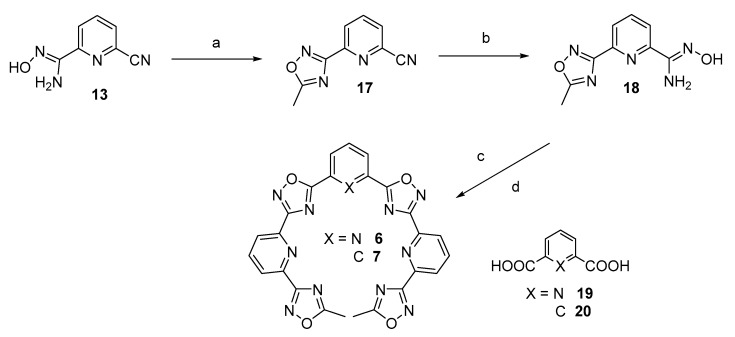
Multi-step synthetic protocol developed for the preparation of the heptaheterocyclic compounds TOxAzaPy and TOxAzaPhen (**6** and **7**): (**a**) acetic anhydride, CHCl_3_, 16 h, r.t.; (**b**) NH_2_OH/Na_2_CO_3_, H_2_O, 1 h, r.t.; (**c**) 1,1’-carbonyldiimidazole (CDI), DMPU, 30 min, r.t.; and (**d**) 1,1’-carbonyldiimidazole (CDI), DMPU, 6 h, 150 °C.

TOxAzaPy (**6**) and TOxAzaPhen (**7**) were synthesized according to the stepwise protocol shown in Scheme 2. The picolin-cyano-monoamidoxime **13** was converted into oxadiazole **17** exploiting a one-pot, two-step synthesis. Compound **13** was firstly *O*-acylated by acetic anhydride and then cycloaromatized in acetic acid to give **17**. Hydroxylamine addition in aqueous ethanol, yielded the formation and the precipitation of the amidoxime **18** as white crystals, directly used in the last step, without further purification. The dicarboxylic acid **19** or **20** was activated to perform the *O*-acylation and the cycloaromatization in a single step, to achieve the final oxadiazoles **6** and **7**. A change in solvent, from DMF to DMPU, implemented the reaction yield of the final cyclization reaction (Table 2).

### 2.2. Screening of the Ligands

The ability of the oligoheteroaryl oxadiazole/pyridine-compounds **1**–**7** (Figure 2) to bind and stabilize G-quadruplex structures was evaluated by performing two well-known biophysical assays, namely FRET melting and G4-FID assay.

#### 2.1.1. FRET-Melting

The G4 stabilizing properties of the compounds **1**–**7** were evaluated by FRET (Förster resonance energy transfer) melting assay [26]. This assay employs fluorescently doubly labeled (FAM and TAMRA dyes as FRET partners) single stranded oligonucleotides mimicking G4 structures. FRET melting provides an assessment of the stabilization effect produced by the binding of the ligand on a G4 structure by measuring the change in the melting temperature (ΔT_m_). In detail, the stabilization is measure as the difference of melting temperature in the presence and in the absence of ligand at the inflection point of the melting curve. First, the experiment was carried out in the presence of F21T (0.2 µM), a 21-mer oligonucleotide mimicking the human telomeric sequence, in both Na^+^- and K^+^-rich buffer and the polyheteroaryl oxadiazole/pyridine-compounds **1**–**7** at the fixed concentration of 1 µM.

**Figure 3:**
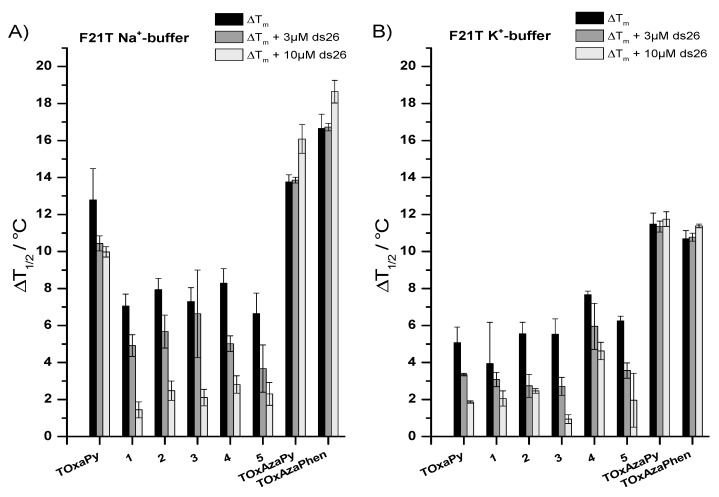
Quantitative analysis of the FRET-melting competition experiments with the telomeric sequence F21T (0.2 µM). Stabilization in: (**A**) Na-rich buffer; or (**B**) K-rich buffer is indicated for the reference compound TOxaPy, in the absence (black bars) or in the presence of double-stranded DNA (ds26) at 3 µM (dark grey bars) or 10 µM (fair grey bars). Analysed compounds: **1**–**5**, TOxAzaPy (**6**) and TOxaAzaPhen (**7**) (1 µM). Error bars corresponds to SD of three independent experiments. Experiments were performed in: (**A**) 10 mM lithium cacodylate buffer (pH 7.2), 100 mM NaCl; and (**B**) 10 mM lithium cacodylate buffer (pH 7.2), 90 mM LiCl, and 10 mM KCl.

All the newly synthesized compounds were able to stabilize F21T and the stabilization was higher in Na^+^- than in K^+^-rich buffer. The ΔT_m_ values of F21T for the penta-aryl compounds **1**–**5** ranged from 6.6 to 8.3 °C in Na^+^-rich buffer and from 3.9 to 7.7 °C in K^+^-rich buffer (Figure 3). Following the same trend, the hepta-aryl ligands TOxAzaPy and TOxAzaPhen showed much higher stabilization ability than the penta-aryl counterparts, with ΔT_m_ values ranging from 13.8 to 16.6 °C in Na^+^-rich buffer (Figure 3A) and from 10.8 to 11.5 °C in K^+^-rich buffer (Figure 3B), respectively. This trend of higher stabilization in Na^+^-rich buffer was previously observed for the hepta-aryl TOxaPy [21], used as a reference compound in this study, although in this specific case the differences in stabilization in the two buffer conditions are less pronounced. As suggested for TOxaPy, these results point out the unusual behavior of these heteroaryl compounds to preferentially recognize and stabilize the antiparallel form of the telomeric sequence (predominantly formed in Na^+^-buffer) more than the [3 + 1] hybrid conformation adopted in K^+^-rich buffer. Based on these results, we decided to analyze our compounds with additional FRET melting studies in the presence of the highly polymorphic telomeric sequence.

To check the selectivity of these ligands towards G4 structures vs. ds DNA, competition FRET melting experiments were carried out in the presence of a large excess of a not-fluorescently labeled double stranded DNA (ds26). This experiment was performed in the classical conditions for FRET melting in the presence of F21T (0.2 µM), ligands (1 µM), and ds26 (either at 3 µM or 10 µM) in Na^+^- or K^+^-rich buffer, respectively (Figure 3). The results showed that penta-aryl compounds **1**–**5** were strongly affected by the presence of the competitor in both buffer conditions. In particular, in the presence of 10 µM ds26 (50 mol. eq. competitor), the stabilizing effect towards F21T was almost erased. Differently, the ability to stabilize G4 structure by hepta-aryl TOxAzaPy and TOxAzaPhen was not affected by the presence of a large excess of ds-competitor, making them highly selective G4 ligands. From the point of view of the selectivity, both the newly made neutral ligands TOxAzaPy and TOxAzaPhen appeared to be much superior to TOxaPy in both Na^+^- and K^+^-rich buffered conditions. These outcomes support the hypothesis that the minimal number of aromatics constituting the oligo-heteroaryl ligands to achieve both high affinity and selectivity toward G4s has to be higher than five. Additionally, a comparison between TOxaPy vs. TOxAzaPy and TOxAzaPhen melting data suggests that the replacement of the 1,3-oxazoles by the 1,2,4-oxadiazole moiety positively affect both G4 affinity and G4 vs. ds selectivity.

**Figure 4:**
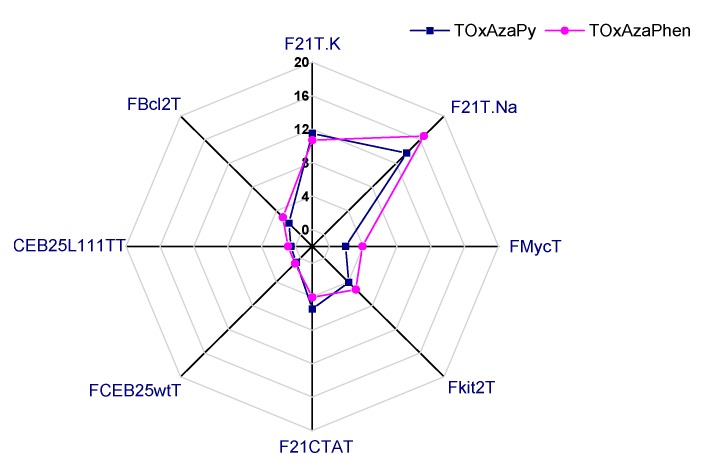
Stabilization of compounds TOxAzaPy and TOxAzaPhen towards several G4 structures. ΔT_m_ values are plotted for each sequence in the buffer conditions mentioned in Table 3.

The stabilization effect of the selective hepta-aryls TOxAzaPy and TOxAzaPhen was challenged towards other G4-forming sequences able to form G-quadruplex structures with different topologies. Moreover, among the parallel conformations, several sequences with different central loop sizes were analyzed. Besides F21T, we selected six additional sequences suitable for FRET melting experiments: parallel forming sequences such as c-myc oncogene promoter (FMycT) [5]; c-kit2 oncogene promoter (Fkit2T) [27]; human minisatellite repeats—native sequence CEB25wt (FCEB25wtT) [28]; modified sequence CEB25L111T (FCEB25L111TT) [29]; Bcl2 proto-oncogene promoter (FBcl2T) [30]; and an antiparallel forming sequence from the human telomeric sequence variant 22CTA (F21CTAT) [31]. FMycT sequence was chosen based on previous experiments performed on oxazole-heteroaryls [23,24]. The idea was to evaluate the effect of the nature of the heterocycle (1,3-oxazole vs. 1,2,4-oxadiazole) on the stabilization of this sequence. TOxAzaPy and TOxAzaPhen show very low stabilization effect towards this sequence, with ΔT_m_ values 1.9 and 3.9 °C, respectively (Table 3 and Figure 4). Similar values were observed with the oxazole family [23,24], suggesting that these oligo-heterocycles are poor ligands for c-myc. By observing the ΔT_m_ values measured in the presence of the two hepta-aryl compounds (Table 3 and Figure 4) it is possible to remark the striking preferential binding towards the human telomeric sequence in both Na^+^- and K^+^-buffer, the moderate stabilization of c-kit2 and 22CTA, and the total lack of stabilization for all the other sequences. Such an apparent selectivity may be due to the highly polymorphism of the telomeric sequence with several structures in equilibrium in solution, which are less stable than the other G4 structures evaluated. In other words, the ligands might have a better chance to adapt to a conformationally flexible target such as 22AG.

The remarkable difference of binding toward F21T in Na^+^ vs. F21CTAT, which share the same topology, by both TOxAzaPy and TOxAzaPhen suggests that TOxAzaPhen and to lower extent TOxAzaPy are structure selective ligands for the antiparallel wild type human telomeric sequence.

#### 2.1.2. G4-FID

According to literature data, BOxAzaPy (Figure 1) and other penta-aryl compounds [22] bind the G4 through a combination of groove binding and π-stacking interactions independently of the structure of the target. The hepta-aryl TOxaPy showing a more elongated structure have been suggested to bind according to a groove binding mode [21]. Although it is likely to expect a similar biding mode for both TOxAzaPy and TOxAzaPhen, the structural selectivity for the antiparallel human telomeric sequence suggests an additional investigation on the binding modality.

**Figure 5:**
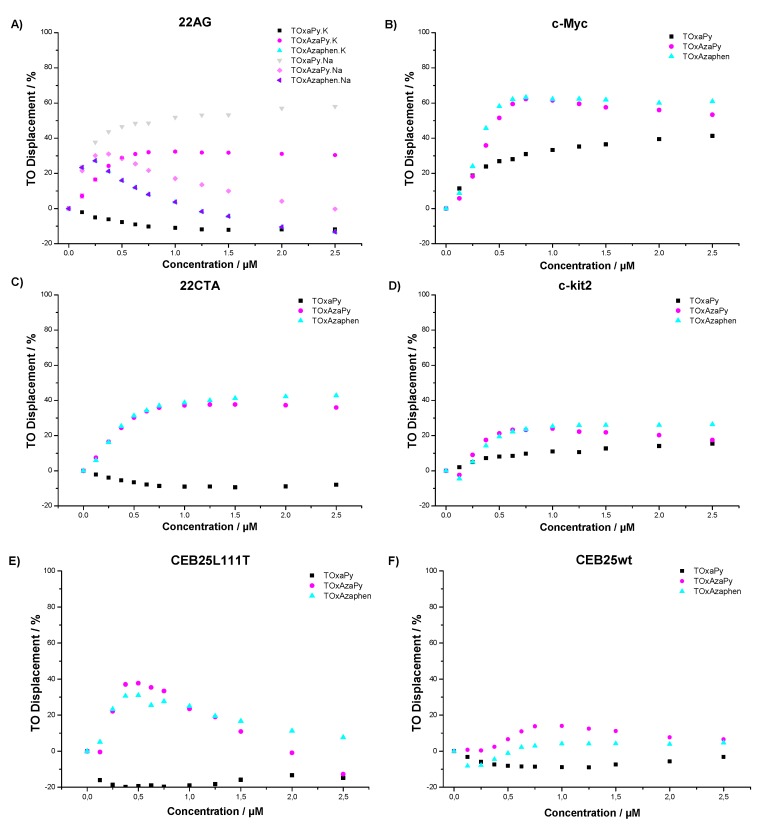
G4-FID plots of TOxAzaPy and TOxAzaPhen in the presence of: (**A**) 22AG.Na and 22AG.K; (**B**) c-Myc; (**C**) 22CTA; (**D**) c-kit2; (**E**) CEB25wt; and (**F**) CEB25L111T. TOxaPy is used as a reference compound.

To gain understanding on the binding modality of TOxAzaPy and TOxAzaPhen, G4-FID titrations were performed. Concisely, this assay is based on the displacement of the fluorescence probe TO (thiazole orange), which binds with no specificity several oligonucleotide structures. In this context, the assay was performed in the presence of six G4 sequences (22AG in both Na^+^- and K^+^-buffers, called 22AG.Na and 22AG.K, respectively; c-myc; c-kit2, 22CTA; CEB25wt; and CEB25L111T) and a duplex DNA (ds26) as a control. We measured TO displacement by TOxAzaPy, TOxAzaPhen and TOxaPy in the presence of the G4 sequences previously mentioned, using the latter as reference compound (Figure 5). The curves of both compounds never reach 100% TO displacement; either they reach saturation a low percentage values (the probe is displaced by up to 40–60% in the best cases with 22CTA and c-myc), or they display complex shape curves (22AG.Na, 22AG.K, c-kit2, and CEB25L111T) suggesting complex equilibrium processes, or they exhibit no TO displacement (CEB25wt). As previously mentioned for this class of compounds [21,22], this kind of curves may reflect a low affinity of the ligands for the TO binding sites and/or an indirect competition between the ligands and TO as a result of binding to different sites of the G4 structure. The indirect competition is caused by the morphological changes onto the G4 structure triggered by the ligand-binding. This competition may cause partial TO displacement or TO re-localization in a site where its fluorescence is brighter than the original one, or can create a new and different TO environment. The re-localization might explain the decreasing in the displacement curves [21]. At last, FID curves in the presence of ds26 (Figure S1) show no binding to duplex DNA. Interestingly, the two new compounds TOxAzaPy and TOxAzaPhen behave differently in comparison to TOxaPy. Due to the similar hepta-heteroaromatic structure of the ligands, we expected a comparable behavior in the TO displacement. Surprisingly we obtained very different results showing that the nature of the penta-aromatic ring has an effect on the binding mode of the ligand towards the selected G4 structures.

The flexible nature of these compounds makes them suitable for loop and groove interactions, as previously described for the structural related TOxaPy [21] and BOxAzaPy derivatives [22], or a mixed binding mode as reported from structural studies obtained for distamycin A [36]. Remarkably, by analyzing FID data, we were able to determine interactions between G4s and both TOxAzaPy and TOxAzaPhen that were not detected by FRET melting assay. A striking example is represented by the c-myc binding, where no stabilization was measured by FRET melting in the presence of both TOxAzaPy and TOxAzaPhen, whereas TO was displaced up to 60%. These results support the idea that FRET melting and G4 FID assays are two complementary biophysical methods that should be used in concert while analyzing the binding and the affinity of G4 ligands towards G4 structures.

### 2.3. CD Titrations

**Figure 6:**
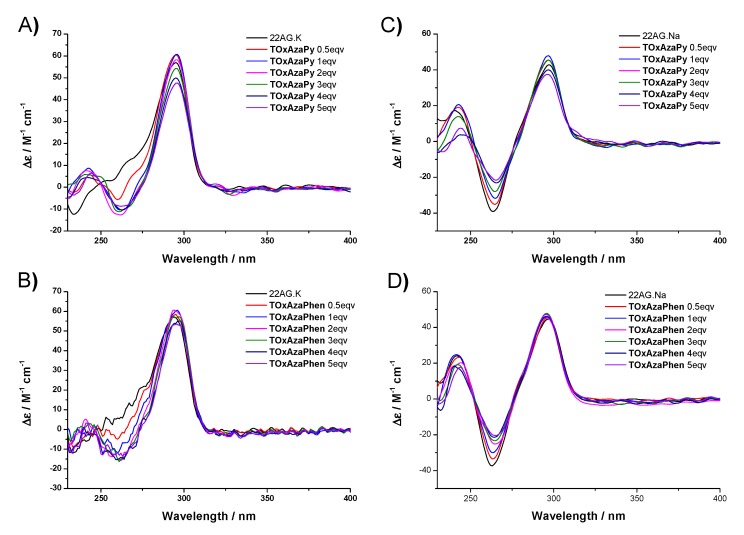
CD spectra of 22AG 5 μM alone and upon addition of increasing concentration of: (**A**) TOxAzaPy in 10 mM lithium cacodylate buffer (pH 7.2), 100 mM KCl; (**B**) TOxAzaPhen in 10 mM lithium cacodylate buffer (pH 7.2), 100 mM KCl; (**C**) TOxAzaPy in 10 mM lithium cacodylate buffer (pH 7.2), 100 mM NaCl; and (**D**) TOxAzaPhen in 10 mM lithium cacodylate buffer (pH 7.2), 100 mM NaCl.

To gain insights into the binding event, highlighting the morphological changes induced by ligand binding to the chiral G4 structures, circular dichroism (CD) experiments were conducted. Due to the higher stabilization observed by FRET melting assay in the presence of TOxAzaPy and TOxAzaPhen, we selected the human telomeric sequence 22AG and we performed CD titrations in both Na^+^- and K^+^-buffer. The CD spectrum of the pre-folded 22AG alone in K^+^-rich buffer shows the characteristic signature reported in the literature for this sequence, displaying a maximum at 290 nm, a shoulder around 265 nm, and a minimum at 235 nm, corresponding to a [3 + 1] hybrid structure [33,34,35]. After addition of TOxAzaPy, an increase of the band at 290 nm together with a conversion of the positive band a 265 nm to negative was observed, suggesting a TOxAzaPy:22AG.K complex formation. This signature is characteristic of an antiparallel structure induced by the presence of the ligand. This trend is observed up to 1 eq. of TOxAzaPy, with the generation of two isoelliptical points at 285 nm and 250 nm suggesting a single equilibrium converting the free DNA into a bound species (Figure 6A). Increasing concentrations of ligand produced a dropping of the intensity of the band a 290 nm, a stabilization of the intensity of the band a 265 nm, and the disappearance of the two isoelliptical points suggesting multiple equilibriums. A very similar behavior was observed during the CD titration performed in the presence of TOxAzaPhen (Figure 6B) with the only difference that the higher molar ellipticity value at 290 nm and the lower value at 265 nm were reached at 2 eq. of ligand. A similar conformational change was induced on 22AG by the cationic BOxAzaPy (Figure 1) [22].

The same experiments were carried out in the presence of 22AG in Na^+^-rich buffer. As reported in the literature, the main conformation generated by the telomeric sequence in this condition is an antiparallel structure characterized by positive bands at 295 nm and 245 nm and a negative band at 265 nm [32,37]. Upon addition of TOxAzaPy, an increase in intensity of the bands at 295 nm, 265 nm, and 245 nm together with a 3 nm hypochromic shift at 295 nm and a 3 nm bathochromic shift at 265 nm was recorded. The first equivalent of ligand induced an increase of the intensity of the bands at 295 nm and 245 nm, differently higher ligand concentrations reduced the band intensities to lower values as compared to the initial ellipticity together with a 6 nm bathochromic shift at 245 nm. Increasing concentration of ligand produced a continuous increasing of the intensity of the band at 265 nm (Figure 6C). Two neat isoelliptical points are visible at 275 nm and 285 nm and a not clear isoelliptical point around 250 nm. TOxAzaPhen showed a similar behavior (Figure 6D) with a mild increase in intensity of the band at 295 nm, a strong increase at 265 nm, and a strong decrease at 245 nm. Two clear isoelliptical points are visible at 275 nm and at 285 nm and a not clear isoelliptical point around 255 nm.

In Na^+^-buffer, an apparent two-state binding mode can be inferred by plotting the variation in intensity of the 292 and 260 nm bands in function of TOxAzaPy and TOxAzaPhen concentration (Figure S2). Global fitting by using non-linear regression of scatter plot at 260 nm—saturation is reached faster at 292 nm giving bigger data fluctuation—with Hill1 equation was achieved, and yielded apparent dissociation constants *K*_d_ = 3.6 × 10^−6^ M (R^2^ = 0.98) and 3.3 × 10^−6^ M (R^2^ = 0.88), for TOxAzaPy and TOxAzaPhen, respectively.

The titration experiments carried out in the presence of TOxAzaPy and TOxAzaPhen and the telomeric sequence both in K^+^- and Na^+^-rich buffer show the generation of hepta-aryls:22AG complexes. In the presence of K^+^, the ligand binding causes a modification of the spectra shape due to a shift of the equilibrium towards conformations exhibiting antiparallel dichroic features. Comparable transitions have been reported for a small number of structurally related oligoaryls [22,23]. In the presence of Na^+^, the dichroic changes suggest a strong interaction without substantial structural transitions. Summing up, both the ligands TOxAzaPy and TOxAzaPhen exhibit a remarkable preference for the human telomeric sequence in the antiparallel conformation.

## 3. Materials and Methods

### 3.1. General Information

All chemicals and solvents were purchased from Sigma Aldrich (Milan, Italy) and used without further purification. Melting points were taken on an Olympus Bx41 polarization microscope apparatus and are uncorrected. TLC analysis was carried out on silica gel (Merck 60F 254, Milan, Italy) with visualization at 254 and 366 nm. Flash chromatography was performed with silica gel 60 (40–63 µm, Merk, Milan, Italy). Biotage (MPLC) Isolera ONE (Biotage AB, Uppsala, Sweden), an automated flash purification system with variable wavelength detector, was performed with SNAP 10 g, 50 g, and 100 g Column, KP-SIL Pk 20, Biotage. All anhydrous reactions were carried out under positive pressure of nitrogen or argon. Elemental analyses were provided by Carlo Erba CHN analyzer (Milan, Italy). All ^1^H-NMR and ^13^C-NMR spectra were recorded on a Bruker Advance 300 MHz spectrometer (Billerica, MA, USA) using deuterated solvents and TMS as internal standard. The spectra are reported in ppm and referenced to deuterated DMSO (2.49 ppm for ^1^H, 39.5 ppm for ^13^C) or deuterated chloroform (7.26 ppm for ^1^H, 77 ppm for ^13^C). The following abbreviations are used: singlet (s), doublet (d), triplet (t) and multiplet (m). Naked and modified oligonucleotide sequences were purchased from Eurogentec (Liege, Belgium). FRET-melting assay were performed in 96-well plates on real time PCR apparatus 7900HT Fast Real-Time PCR System (Abi Applied Biosystems, Waltham, MA, USA). G4-FID assays were performed in a fluorescence Agilent Cary Eclipse spectrophotometer (Agilent Technologies, Santa Clara, CA, USA), and a temperature of 20 °C was kept constant with thermostated cell holders (1 mL reaction volume). CD experiments were carried out at 20 °C with a JASCO J-710 spectropolarimeter (JASCO corporation, Easton, MD, USA) equipped with a Peltier temperature controller (Jasco PTC-348WI, JASCO corporation, Easton, MD, USA) interfaced to a PC, by using 1 cm path rectangular quartz cells (1 mL reaction volume).

### 3.2. Synthetic Methods

#### 3.2.1. General Procedure for the Synthesis of **1**–**4**

The correspondent carboxylic acid (3.4 mmol) and 1,1′-carbonyldiimidazole (CDI, 3.4 mmol) were dissolved in DMF (15 mL) and stirred at room temperature for 30 min. (2*Z*, 6*Z*)-*N*′2,*N*′6-Dihydroxypyridine-2,6 bis(carboximidamide) (1.53 mmol) was added and the reaction mixture was stirred at room temperature overnight. CDI (3.4 mmol) was further added and the reaction mixture was heated at 150 °C for 6 h. After cooling down, the reaction mixture was poured into water to induce the precipitation of a solid, purified by Isolera ONE (CHCl_3_:MeOH gradient 0 to 20%), which was filtered and characterized as pure product.

*2,6-Bis(5-(furan-2-yl)-1,2,4-oxadiazol-3-yl)pyridine* (**1**). White solid, Yield: 11 %, m.p. > 300  °C (dec.); ^1^H-NMR (300 MHz, CDCl_3_, 25 °C, TMS): δ = 8.37 (d, 3*J*(H,H) = 7.8 Hz, 2 H), 8.10 (t, 3*J*(H,H) = 8 Hz, 1 H), 7.77 (d, 3*J*(H,H) = 1.2 Hz, 2 H), 7.51 (d, 3*J*(H,H) = 3.6 Hz, 2 H), 6,7 (dd, 3*J*(H,H) = 3.5 Hz; 3*J*(H,H) = 1.6 Hz, 2 H). ^13^C-NMR (75 MHz, CDCl_3_, 25 °C, TMS): δ = 168.3, 167.8, 146.9, 146.8, 139.8, 138.3, 125.1, 117.3, 112.6; elemental analysis calcd. (%) for C_17_H_9_N_5_O_4_: C, 58.79; H, 2.61; N, 20.17; found C, 57.98; H, 2.70; N, 20.07.

*2,6-Bis(5-(thiophen-2-yl)-1,2,4-oxadiazol-3-yl)pyridine* (**2**). White solid, Yield: 11 %, m.p. > 300  °C; ^1^H-NMR (300 MHz, CDCl_3_, 25 °C, TMS): δ = 8.46 (d, 3*J*(H,H) = 7.8 Hz, 2 H), 8.19 (t, 3*J*(H,H) = 7.8 Hz, 1 H), 8.17 (dd, 3*J*(H,H) = 2.4 Hz, 3*J*(H,H) = 1.3 Hz 2 H), 7.82 (dd, 3*J*(H,H) = 5 Hz, 3*J*(H,H) = 1.05 Hz, 2 H), 7.36 (dd, 3*J*(H,H) = 4.9 Hz, 3*J*(H,H) = 3.8 Hz, 2 H). ^13^C-NMR (75 MHz, CDCl_3_, 25 °C, TMS): δ = 172.2, 167.9, 146.9, 138.2, 132.5, 132.4, 128.5, 125.3, 124.9; elemental analysis calcd. (%) for C_17_H_9_N_5_O_2_S_2_: C, 53.81; H, 2.39; N, 18.46; found C, 53.12; H, 2.51; N, 19.02.

*2,6-Bis(5-(benzofuran-2-yl)-1,2,4-oxadiazol-3-yl)pyridine* (**3**). White solid, Yield: 28 %, m.p. > 300  °C; ^1^H-NMR (300 MHz, CDCl_3_, 25 °C, TMS): δ = 8.44 (d, 3*J*(H,H) = 7.8 Hz, 2 H), 8.16 (t, 3*J*(H,H) = 7.8 Hz, 1 H), 7.88 (s, 2 H), 7.80 (d, 3*J*(H,H) = 7.7 Hz, 2 H), 7.72 (d, 3*J*(H,H) = 8.4 Hz, 2 H), 7.55 (t, 3*J*(H,H) = 7.5 Hz, 2 H), 7.41 (t, 3*J*(H,H) = 7.5 Hz, 2 H). ^13^C-NMR (75 MHz, CDCl_3_, 25 °C, TMS): δ = 168.5, 167.7, 146.4, 140.3, 138.0, 127.6, 126.6, 126.1, 124.9, 123.9, 122.4, 113.1, 111.9; elemental analysis calcd. (%) for C_25_H_13_N_5_O_4_: C, 67.11; H, 2.93; N, 15.65; found C, 57.01; H, 2.77; N, 15.02.

*2,6-Bis(5-(1H-indol-2-yl)-1,2,4-oxadiazol-3-yl)pyridine* (**4**). White solid, Yield: 10%, m.p. > 300  °C; ^1^H-NMR (300 MHz, CDCl_3_, 25 °C, TMS): δ = 9.49 (s, 2 H), 8.38 (d, 3*J*(H,H) = 7.7 Hz, 2 H), 8.10 (t, 3*J*(H,H) = 7.3 Hz, 1 H), 7.77 (d, 3*J*(H,H) = 7.7 Hz, 2 H), 7.52 (s, 2 H), 7.40 (t, 3*J*(H,H) = 7.2 Hz, 2 H), 7.31 (m, 4 H). elemental analysis calcd. (%) for C_25_H_15_N_7_O_2_: C, 67.41; H, 3.39; N, 22.01; found C, 67.12; H, 3.51; N, 21.82.

#### 3.2.2. Stepwise Synthesis for (**5**)

*6-(5-(Quinolin-2-yl)-1,2,4-oxadiazol-3-yl)picolinonitrile* (**15**). Quinoline-2-carboxylic acid (0.12 g, 0.68 mmol) and 1,1′-carbonyldiimidazole (CDI, 0.11 g, 0.68 mmol) were dissolved in DMF (10 mL) and stirred at room temperature for 30 min. (*Z*)-6-cyano-*N*′-hydroxypicolinimidamide (0.1 g, 0.62 mmol) was added and the reaction mixture was stirred at room temperature overnight. CDI (0.11 g, 0.68 mmol) was further added and the reaction mixture was heated at 150 °C for 6 h. After cooling down, the reaction mixture was poured into water to induce the precipitation of a pale yellow solid, purified by Isolera ONE (CHCl_3_:MeOH gradient) obtaining a white solid (0.11 g; yield 60%), which was filtered and characterized as pure product. m.p. > 300  °C (dec.); ^1^H-NMR (300 MHz, [D6]DMSO, 25 °C, TMS): δ = 8.73 (d, 3*J*(H,H) = 8.5 Hz, 1 H), 8.53 (d, 3*J*(H,H) = 7.8 Hz, 1 H), 8.43 (d, 3*J*(H,H) = 7.8 Hz, 1 H), 8.36 (t, 3*J*(H,H) = 7.8, 1 H), 8.27 (d, 3*J*(H,H) = 8.4 Hz, 2 H), 8.17 (d, 3*J*(H,H) = 7.9 Hz, 1 H), 7.96 (t, 3*J*(H,H) = 7.2, 3*J*(H,H) = 8.1 Hz, 1 H), 7.82 (t, 3*J*(H,H) = 7.4, 3*J*(H,H) = 7.6 Hz, 1 H). ^13^C-NMR (75 MHz, [D6]DMSO, 25 °C, TMS): δ = 175.2, 167.5, 147.4, 147.0, 142.6, 139.9, 138.4, 133.4, 131.2, 130.9, 129.7, 128.9, 128.2, 127.1, 120.6, 116.8.

*(Z)-N*′*-Hydroxy-6-(5-(quinolin-2-yl)-1,2,4-oxadiazol-3-yl)-picolinimidamide* (**16**). A mixture of hydroxylamine hydrochloride (0.047 g, 0.68 mmol) and of Na_2_CO_3_ (0.036 g, 0.34 mmol) in water (10 mL) was added dropwise in 45 min to a solution containing 6-(5-(quinolin-2-yl)-1,2,4-oxadiazol-3-yl)picolinonitrile (0.1 g, 0.33 mmol) in EtOH (30 mL) and the reaction mixture was stirred at r.t. for 1 h. The precipitated white solid (0.095 g, yield 92%) was filtered and dried. m.p. = 225–230 °C; ^1^H-NMR (300 MHz, [D6]DMSO, 25 °C, TMS): δ = 10.09 (s, 1 H), 8.74 (d, 3*J*(H,H) = 8.5 Hz, 1 H), 8.45 (d, 3*J*(H,H) = 8.5 Hz, 1 H), 8.27 (m, 2 H), 8.18 (d, 3*J*(H,H) = 8.2, 1 H), 8.11 (d, 3*J*(H,H) = 4.1 Hz, 2 H), 7.96 (dt, 3*J*(H,H) = 1.4 Hz, 3*J*(H,H) = 8.3, 1 H), 7.82 (t, 3*J*(H,H) = 1.2, 3*J*(H,H) = 8.1 Hz, 1 H), 5.89 (s, 2 H). ^13^C-NMR (75 MHz, [D6]DMSO, 25 °C, TMS): δ = 174.8, 168.3, 150.8, 148.9, 147.3, 144.3, 142.8, 138.4, 138.3, 131.2, 129.7, 129.1, 128.9, 128.3, 123.7, 121.8, 120.6.

*2,6-Bis(5-(quinolin-2-yl)-1,2,4-oxadiazol-3-yl)pyridine* (**5**). Quinoline-2-carboxylic acid (0.126 g, 0.72 mmol) and 1,1′-carbonyldiimidazole (CDI, 0.12 g, 0.72 mmol) were dissolved in DMF (10 mL) and stirred at room temperature for 30 min. (2*Z*, 6*Z*)-*N*′2,*N*′6-Dihydroxypyridine-2,6-bis(carboximidamide) (0.2 g, 0.62 mmol) was added and the reaction mixture was stirred at room temperature overnight. CDI (0.12 g, 0.72 mmol) was further added and the reaction mixture was heated at 150  °C for 6 h. After being cooled, the reaction mixture was poured into water to induce the precipitation of a pale with solid, purified by Isolera ONE (CHCl_3_:MeOH gradient) obtaining a white solid (0.151 g; yield 53 %), which was filtered and characterized as pure product; m.p. > 300  °C (dec.). ^1^H-NMR (300 MHz, [D6]DMSO, 25 °C, TMS): δ = 8.73 (d, 3*J*(H,H) = 8.4 Hz, 2 H), 8.47 (m, 4 H), 8.40 (t, 3*J*(H,H) = 7.1 Hz, 1 H), 8.29 (d, 3*J*(H,H) = 8.3, 2 H), 8.17 (d, 3*J*(H,H) = 7.95 Hz, 2 H), 7.96 (t, 3*J*(H,H) = 7.1 Hz, 3*J*(H,H) = 7.5 Hz, 2 H), 7.82 (t, 3*J*(H,H) = 6.9, 3*J*(H,H) = 7.3 Hz, 1 H). HRMS (ESI-MS): 470.1356 (calculated: 470.1365 C_27_H_17_N_7_O_4_^+^); elemental analysis calcd. (%) for C_27_H_15_N_7_O_2_: C, 69.08; H, 3.22; N, 20.89; found C, 69.18; H, 3.10; N, 20.13.

#### 3.2.3. Stepwise Synthesis of TOxAzaPy and TOxAzaPhen

*6-(5-Methyl-1,2,4-oxadiazol-3-yl)picolinonitrile* (**17**). (*Z*)-6-cyano-*N*′-hydroxypicolinimidamide (0.3 g, 1.86 mmol), was dissolved in 15 mL of chloroform and treated with acetic anhydride (0.21 mL, 2.22 mmol) and Et_3_N (0.3 mL) and the mixture was stirred overnight at room temperature. The solvent was removed under reduce pressure and the crude product was dissolved in acetic acid and refluxed for 4 h. The reaction mixture was neutralized with a saturated solution of NaHCO_3_. A white solid precipitated was filtered and washed with water (0.23 g, yield 65%). ^1^H-NMR (300 MHz, CDCl_3_, 25 °C, TMS): δ = 8.35 (d, 3*J*(H,H) = 7.2 Hz, 1 H), 8.05 (t, 3*J*(H,H) = 7.8 Hz, 1 H), 7.86 (d, 3*J*(H,H) = 7.8 Hz, 1 H), 2.75 (s, 1 H). ^13^C-NMR (75 MHz, CDCl_3_, 25 °C, TMS): δ = 179.8, 168.7, 149.7, 140.1, 136.3, 131.7, 127.8, 118.1, 14.2.

*(Z)-N*′*-Hydroxy-6-(5-methyl-1,2,4-oxadiazol-3-yl)picolinimidamide* (**18**). An aqueous solution (30 mL) containing hydroxylamine hydrochloride (0.150 g, 1.1 mmol), and sodium carbonate (0.116 g, 1.08 mmol) was added to a solution of 6-(5-methyl-1,2,4-oxadiazol-3-yl)picolinonitrile **17** (0.2 g, 1.08 mmol) in ethanol (50 mL). The hydroxylamine was added dropwise in 30 min and then the mixture was stirred for further 1 h. The precipitated white solid (0.21 g, yield 89%) was filtered, dried, and used without any further purification. ^1^H-NMR (300 MHz, [D6]DMSO, 25 °C, TMS): δ = 10.13 (s, 1 H), 8.04 (m, 3 H), 5.87 (s, 2 H), 2.71 (s, 3 H). ^13^C-NMR (75 MHz, [D6]DMSO, 25 °C, TMS): δ = 177.9, 167.3, 150.5, 148.8, 144.5, 138.2, 123.3, 121.5, 12.07.

#### 3.2.4. General Procedure for the Synthesis of TOxAzaPy-Me (**6**) and TOxAzaPhen (**7**)

The corresponding dicarboxylic acid (0.21 mmol) and 1,1′-carbonyldiimidazole (CDI, 0.46 mmol) were dissolved in DMPU (2 mL) and stirred at room temperature for 30 min. (*Z*)-*N*′-hydroxy-6-(5-methyl-1,2,4-oxadiazol-3-yl)picolinimidamide **18** (0.46 mmol) was added and the reaction mixture was stirred at room temperature overnight. CDI (0.46 mmol) was further added and the reaction mixture was heated at 150 °C for 6 h. After cooling down, the reaction mixture was poured into water to induce the precipitation of a white solid, purified by Isolera ONE (CHCl_3_:MeOH 0 to 5%) obtaining a solid, which was filtered and characterized as pure product.

*2,6-Bis(5-(6-(5-methyl-1,2,4-oxadiazol-3-yl)pyridin-2-yl)-1,2,4-oxadiazol-3-yl)pyridine TOxAzaPy* (**6**). White solid, Yield: 19 %, m.p. > 300  °C (dec.); ^1^H-NMR (300 MHz, [D6]DMSO, 25 °C, TMS): δ = 8.68 (d, 3*J*(H,H) = 7.8 Hz, 2 H), 8.49 (t, 3*J*(H,H) = 8.1 Hz, 1 H), 8.41 (dd, 3*J*(H,H) = 6.2 Hz, 3*J*(H,H) = 2.7 Hz, 2 H), 8.31–8.27 (m, 4 H), 2.74 (s, 3 H). ^13^C-NMR (75 MHz, [D6]DMSO, 25 °C, TMS): δ = 177.9, 174.1, 168.5, 167.4, 146.8, 146.2, 143.6, 140.4, 139.4, 127.5, 125.4, 11.9; elemental analysis calcd. (%) for C_25_H_15_N_11_O_4_: C, 56.29; H, 2.83; N, 28.88; found C, 56.93; H, 2.92; N, 29.02.

*1,3-Bis(5-(6-(5-methyl-1,2,4-oxadiazol-3-yl)pyridin-2-yl)-1,2,4-oxadiazol-3-yl)benzene TOxAzaPhen* (**7**). White solid, Yield: 38 %, m.p. > 300 °C (dec.); ^1^H-NMR (300 MHz, CDCl_3_, 25 °C, TMS): δ = 9.22 (s, 1 H), 8.56 (d, 3*J*(H,H) = 7.5 Hz, 2 H), 8.39 (d, 3*J*(H,H) = 7.8, 2 H), 8.29 (d, 3*J*(H,H) = 7.7 Hz, 2 H), 8.09 (t, 3*J*(H,H) = 7.8 Hz, 2 H), 7.82 (t, 3*J*(H,H) = 7.8 Hz, 1 H), 2.75 (s, 6 H). ^13^C-NMR (75 MHz, CDCl_3_, 25 °C, TMS): δ = 177.9, 174.1, 168.5, 167.4, 146.8, 146.2, 143.6, 140.4, 139.4, 127.5, 125.4, 11.9; elemental analysis calcd. (%) for C_26_H_16_N_10_O_4_: C, 58.65; H, 3.03; N, 26.31; found C, 58.12; H, 3.21; N, 26.08.

### 3.3. Biophysical Assays

#### 3.3.1. FRET Melting Assay

Stabilization of compounds with quadruplex-structure was monitored via FRET-melting assay performed in 96-well plates on real time PCR apparatus 7900HT Fast Real-Time PCR System as follow: 5 min at 25 °C, then increase of 0.5 °C every minute until 95 °C. Each experimental condition was tested in duplicated in a volume of 25 µL for each sample. FRET-melting assay was performed with oligonucleotides that mimic the human telomeric sequence, as well as other quadruplex-forming oligonucleotides, equipped with FRET partners (here: FAM and TAMRA) at each extremity in the presence of the G4 ligand. To determine ligand G4 vs. duplex selectivity, an unlabeled DNA competitor ds26 was used. The oligonucleotides were prepared at 0.2 µM, the ligands at 1 µM, and competitors at 0, 3, and 10 µM final concentration. Measurements were made with excitation at 492 nm and detection at 516 nm in a buffer of lithium cacodylated (10 mM, pH 7.2), NaCl (100 mM) or KCl (10 mM, completed by 90 mM LiCl for F21T, and 1 mM, completed by 99 mM LiCl for all the others G-quadruplex sequences). To identify if the ligands are able to stabilize the G-quadruplex structure, we determined the temperature at half denaturation of the G4 in the absence and in the presence of ligand. The experiments were carried out on prefolded G-quadruplex structures: the sequences were heated at 90 °C for 5 min and left to cool down at 4 °C overnight. At last, to identify a preferential binding towards a particular G-quadruplex conformation, a series of labeled oligonucleotides covering a range of possible G4 conformations was used (Table 4):

#### 3.3.2. G4-FID Assay

Cell G4-FID assays were performed in a fluorescence Agilent Cary Eclipse spectrophotometer. A temperature of 20 °C was kept constant with thermostated cell holders. Each experiment was performed in a 1 mL cell, in 10 mM lithium cacodylate buffer (pH 7.4) with 100 mM KCl or NaCl depending on the experiment, in a total volume of 1 mL. The G4-FID assay was designed as follows: 0.25 μM pre-folded DNA target was mixed with thiazole orange (0.50 μM for G4-DNA, 0.75 μM for ds26). Each ligand addition (from 0.5 to 10 equivalents) was followed by a 3 min equilibration time, after which the fluorescence spectrum was recorded. The percentage of displacement was calculated as follows: TO displacement (%) = 100 − [(FA/FA_0_) × 100], where FA and FA_0_ stand for the fluorescence emission area of TO bound to DNA after each ligand addition and FA_0_ before ligand addition (area measured from 510 to 750 nm, λexc = 495 nm, slit = 10 nm). The percentage of displacement was then plotted as a function of the concentration of added ligand. As for the FRET melting assay a series of oligonucleotides covering a range of possible G4 conformations was used (Table 5).

#### 3.3.3. Circular Dichroism (CD) Titration

CD experiments were carried out at 20 °C with a JASCO J-710 spectropolarimeter equipped with a Peltier temperature controller (Jasco PTC-348WI) interfaced to a PC, by using 1 cm path rectangular quartz cells (1 mL reaction volume). Scans were recorded from 220 nm to 400 nm with the next parameters: 100 mdeg sensitivity, 1 nm data pitch, 200 nm min^−1^ scan speed, 1 s response, 1 nm band width, and 4 accumulations. Solutions containing 5 μM 22AG were titrated with increasing concentration of ligands (0–5 eq, 10 min stabilization time) in lithium cacodylate (10 mM) supplemented with potassium or sodium chloride (100 mM) at pH 7.2. The scan of the buffer was subtracted from the average of the scan of the samples. The signal was further smoothened with a Savitzky–Golay method (2 order, 20-point window). The CD data were blank-subtracted and normalized to molar dichroic absorption (Δε) on the basis of concentration using Equation (1), with θ the ellipticity in millidegrees, c concentration in mol L^−1^, and l the path length in cm [38]:

Δε = θ/(32980 × c × l)
(1)

## 4. Conclusions

A new series of non-macrocyclic olygoheteroaryls (**1**–**7**) containing 1,2,4-oxadiazoles and carbo- or hero-aromatics were synthesized according to a one-pot, two-step synthesis or following a more time consuming, but more efficient stepwise protocol. Compounds **1**–**7** were evaluated as ligands towards secondary nucleic acid structures mimicking cancer-associated G-quadruplexes (e.g., 22AG for human telomeric sequence, c-myc, and c-kit promoters). Their binding properties were compared to the structurally related reference compound TOxaPy, by FRET melting, G4-FID assays and circular dichroism. Pentaheteroaryls showed to be very poor G-quadruplex ligands, corroborating previous results on cationic analogs (BOxAzaPy), the binding properties of which were mainly conferred by the charged appendages. On the contrary, more extended heptapyridyl-oxadiazole ligands, such as TOxAzaPy and TOxAzaPhen, showed preferential binding towards the antiparallel telomeric sequence (22AG) with remarkable selectivity vs. duplex DNA. From this point of view, and for potency as well, they compare favorably to TOxaPy as G-quadruplex ligands. Our evidence suggests that 1,2,4-oxadiazole moieties introduced in the G4-ligand as structural modification, implement and ameliorate the G-quadruplex binding properties of the hepta-aryls, which mainly act as groove ligands.

## Figures and Tables

**Table 1:** Penta-heterocyclic compounds.

R	Pathway	Compound	Yield (%)
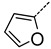	A	**1**	11
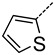	A	**2**	11
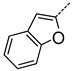	A	**3**	28
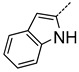	A	**4**	10
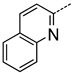	B	**5**	53

**Table 2:** Overall reaction yields of the heptaheterocyclic compounds.

X	Compound	Yield (%)
N	**6**	19
C	**7**	38

**Table 3:** ΔT_m_ values for **6** and **7** (1 µM) towards several G4 structures.

G4 Sequences	Topology	TOxAzaPy ΔT_m_ (°C)	TOxAzaPhen ΔT_m_ (°C)
F21T.Na^+^	Antiparallel [32]	13.8 ± 0.4	16.6 ± 0.8
F21T.K^+^	Hybrid/mix of several topologies [32,33,34,35]	11.5 ± 0.6	10.8 ± 0.4
FMycT	parallel	1.9 ± 0.1	3.9 ± 0.9 ^1^
Fkit2T	parallel	4.1 ± 0.3	5.3 ± 0.4 ^2^
FCEB25wtT	parallel	0.7 ± 0.9	0.9 ± 0.7 ^1^
FCEB25L111TT	parallel	0.5 ± 0.3	0.9 ± 0.8 ^1^
FBcl2T	parallel	1.9 ± 0.7	2.9 ± 0.7 ^2^
F21CTAT	antiparallel	5.5 ± 0.2	4.1 ± 0.1 ^2^

Note: ^1^ 10 mM lithium cacodylate buffer (pH 7.2), 99 mM LiCl, and 1 mM KCl; ^2^ 10 mM lithium cacodylate buffer (pH 7.2), 90 mM LiCl, and 10 mM KCl.

**Table 4:** DNA sequences employed for the FRET melting assay.

Sequence Name	Sequence (5′-3′)
F21T	Fam-G_3_TTAG_3_TTAG_3_TTA G_3_-Tamra
FMycT	Fam-TTGAG_3_TG_3_TAG_3_TG_3_TAA-Tamra
F21CTAT	Fam-G_3_CTAG_3_CTAG_3_CTAG_3_-Tamra
FKit2T	Fam-G_3_CG_3_CGCGAG_3_AG_4_-Tamra
FCEB25wtT	Fam-AAG_3_TG_3_TGTAAGTGTG_3_TG_3_T-Tamra
FCEB25-L111TT	Fam-AAG_3_TG_3_TG_3_TG_3_T-Tamra
FCEB25-L121(AA)TT	Fam-AAG_3_TG_3_AAG_3_TG_3_T-Tamra
FCEB25-L121TT	Fam-AAG_3_TG_3_TTG_3_TG_3_T-Tamra
FBcl2T	Fam-AG_4_CG_3_CGCG_3_AG_2_AAG_5_CG_3_AGCG_4_CTG-Tamra
ds26	CAATCGGATCGAATTCGATCCGATTG

**Table 5:** DNA sequences employed for the G4-FID assay.

Sequence Name	Sequence (5′-3′)
22AG	AG_3_TTAG_3_TTAG_3_TTAG_3_
Myc22	TGAG_3_TG_3_TAG_3_TG_3_TAA
22CTA	AG_3_CTAG_3_CTAG_3_CTAG_3_
c-kit2	G_3_CG_3_CGCGAG_3_AG_4_
CEB25wt	AAG_3_TG_3_TGTAAGTGTG_3_TG_3_T
CEB25-L111T	AAG_3_TG_3_TG_3_TG_3_T
Bcl2	AG_4_CG_3_CGCG_3_AG_2_AAG_5_CG_3_AGCG_4_CTG
ds26	CAATCGGATCGAATTCGATCCGATTG

## Supplementary Materials

The following are available online, LC-MS analysis, ^1^H and ^13^C NMR, Figure S1: G4-FID in the presence of ds26, Figure S2: Titration plots representing the change in ellipticity of 22AG in Na^+^-rich buffer at 292 nm and 260 nm with the addition of TOxAzaPy and TOxAzaPhen.
