# Supplementary material for: Oxadiazole/Pyridine-Based Ligands: A Structural Tuning for Enhancing G-Quadruplex Binding
Source: Molecules. 2018 Aug 28;23(9):2162. doi: 10.3390/molecules23092162 (PMC6225118; doi:10.3390/molecules23092162)
Supplement: Supplementary file 1 [file molecules-23-02162-s001.pdf]

Oxadiazole/pyridine-based ligands: a structural tuning for enhancing G-quadruplex binding

Filippo Doria,[1] Valentina Pirota,[1] Michele Petenzi,[1] Marie-Paule Teulde Fichou,[2] Daniela Verga[2,*] and Mauro Freccero [1,*]

[1] Dipartimento di Chimica, Università di Pavia, Pavia, 27100, Italy; filippo.doria@unipv.it; valentina.pirota01@universitadipavia.it, michele.petenzi@gmail.com.

[2] Institut Curie, Section Recherche, CNRS, UMR 176, Université Paris-Sud, Bat. 110–112, 91405 Orsay, France ; mp.teulade-fichou@curie.fr

* Correspondence: daniela.verga@curie.fr; mauro.freccero@unipv.it; Tel.: +39-0382-987668

Supplementary Material

Table of Contents:

LC-MS Analysis. Pag. S2-S10

NMR Figures. ^{1}H and ^{13}C NMR characterization of all compounds presented in the main text S11-S19

Figure S1: G4-FID in the presence of ds26 S20

Figure S2: Titration plots representing the change in ellipticity of 22AG in Na^{+}-rich buffer at 292 nm and 260 nm with the addition of TOxAzaPy and TOxAzaPhen S21

LC-MS Analysis

LC-MS analysis was performed using a Jasco UPLC-system combined to a Thermo LTQ-Orbitrap XL. The solvents used for all the LC-MS analyses and purifications were 0.1 % Formic acid in water and acetonitrile. The column was Acquity UPLC BEH C18 (1.7Mm) (50 x 2.1 mm) (Waters). The following analytical method was used, flow: 0.3 mL/min; gradient: 95% aqueous, gradually to 100% aqueous over 11 minutes and then isocratic flow for 4 minutes.

Compound **1**

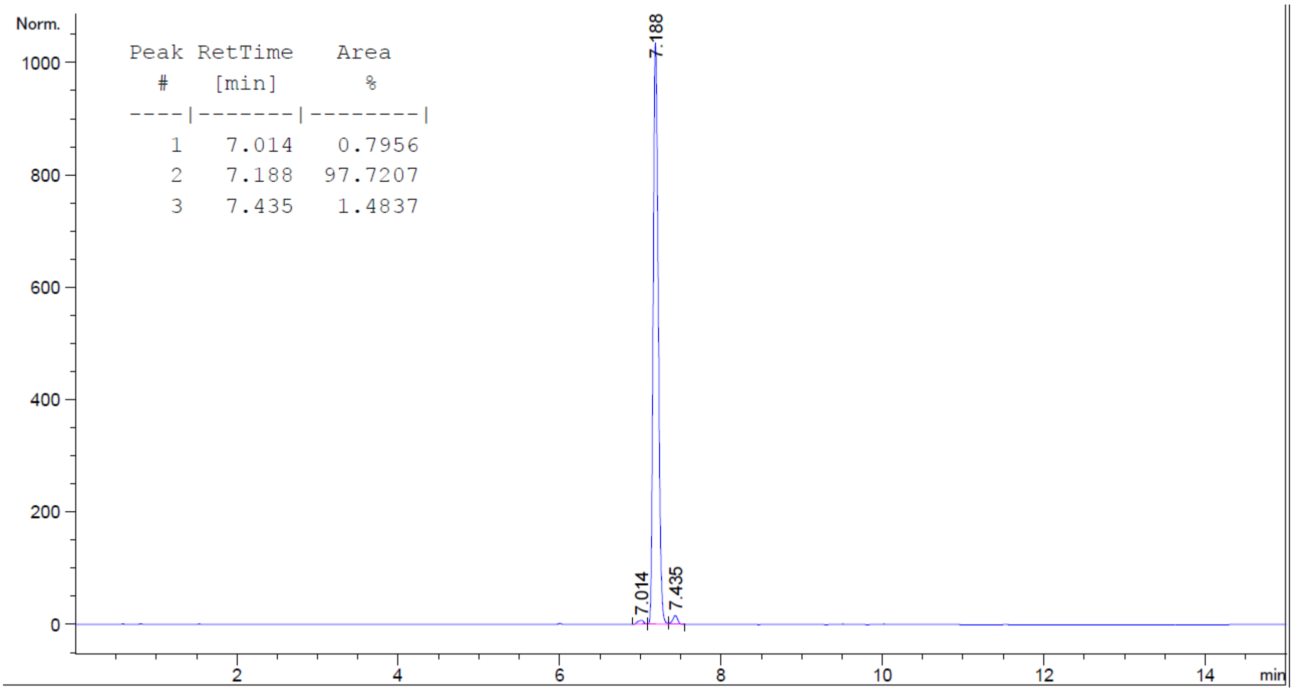

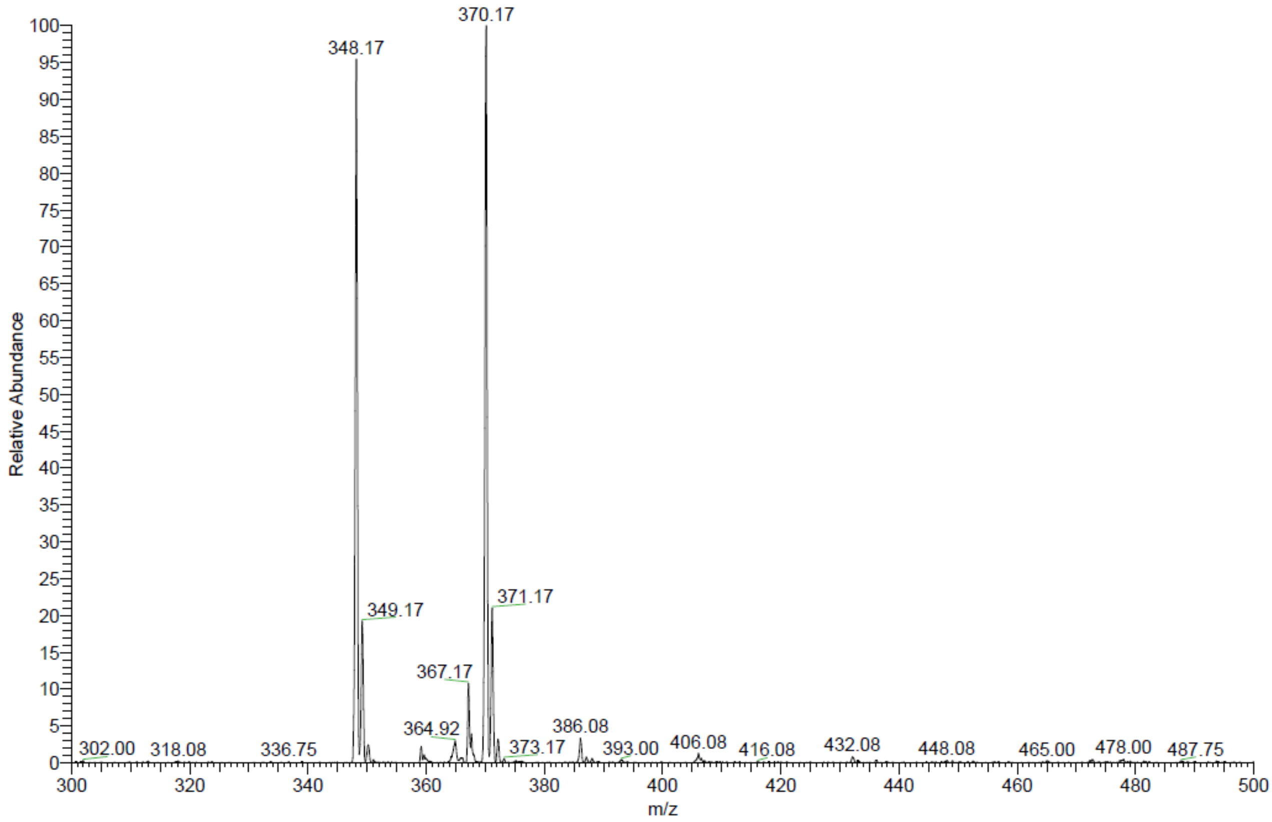

Compound **2**

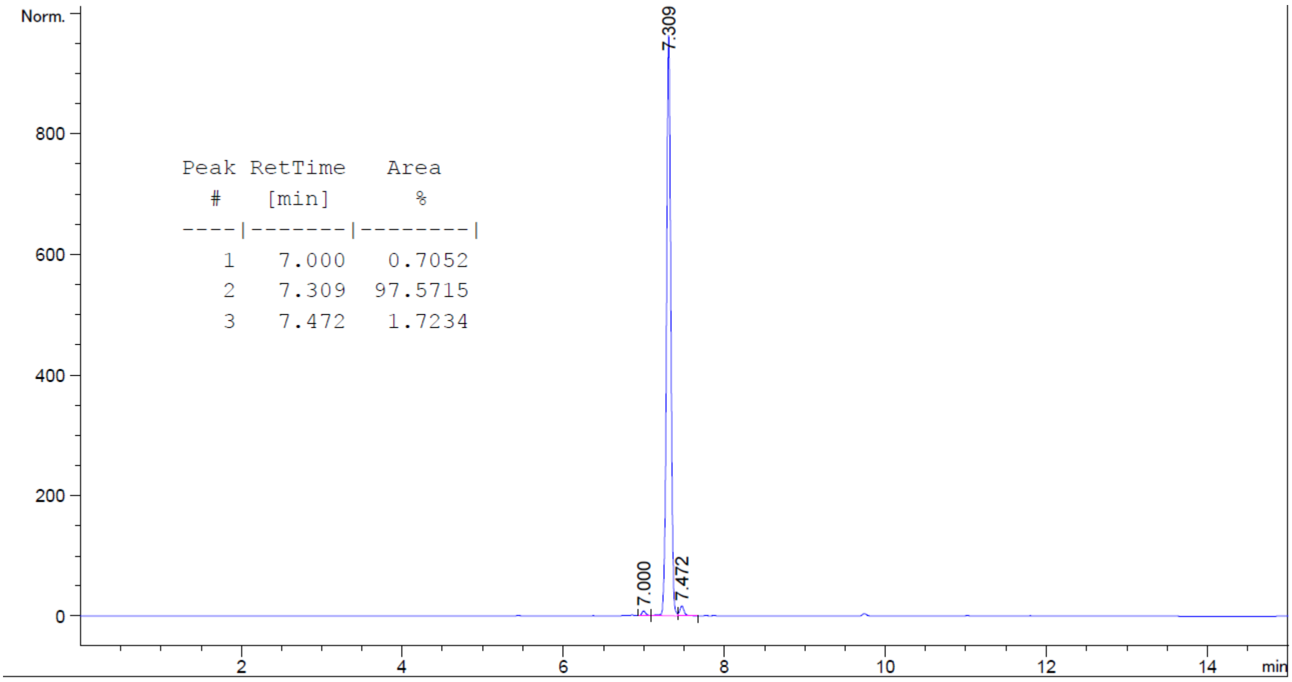
Norm.
800
600
400
200
0
7.309
7.000
7.472
Peak RetTime Area
[min] %
1 7.000 0.7052
2 7.309 97.5715
3 7.472 1.7234
2
4
6
8
10
12
14
min

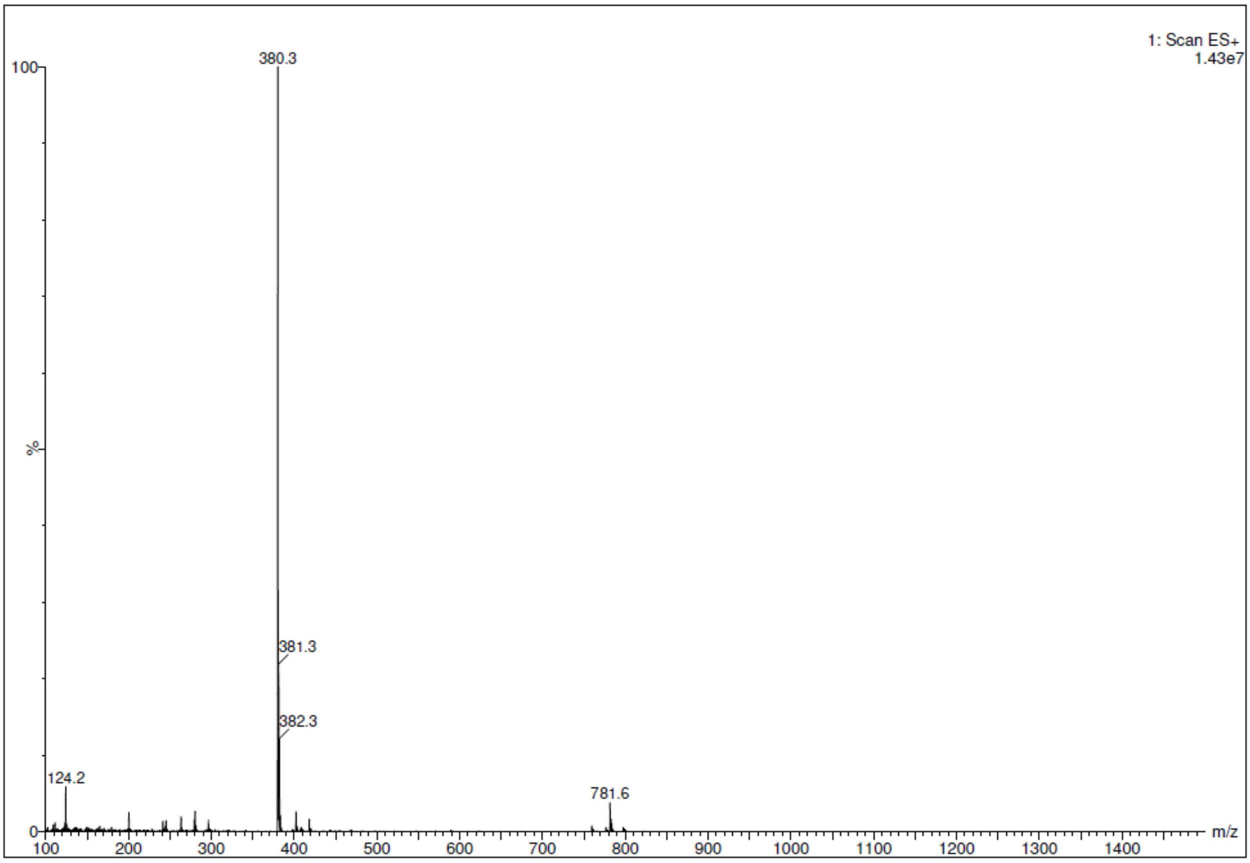
1: Scan ES+
1.43e7
100
%
0
380.3
381.3
382.3
124.2
781.6
m/z

Compound **3**

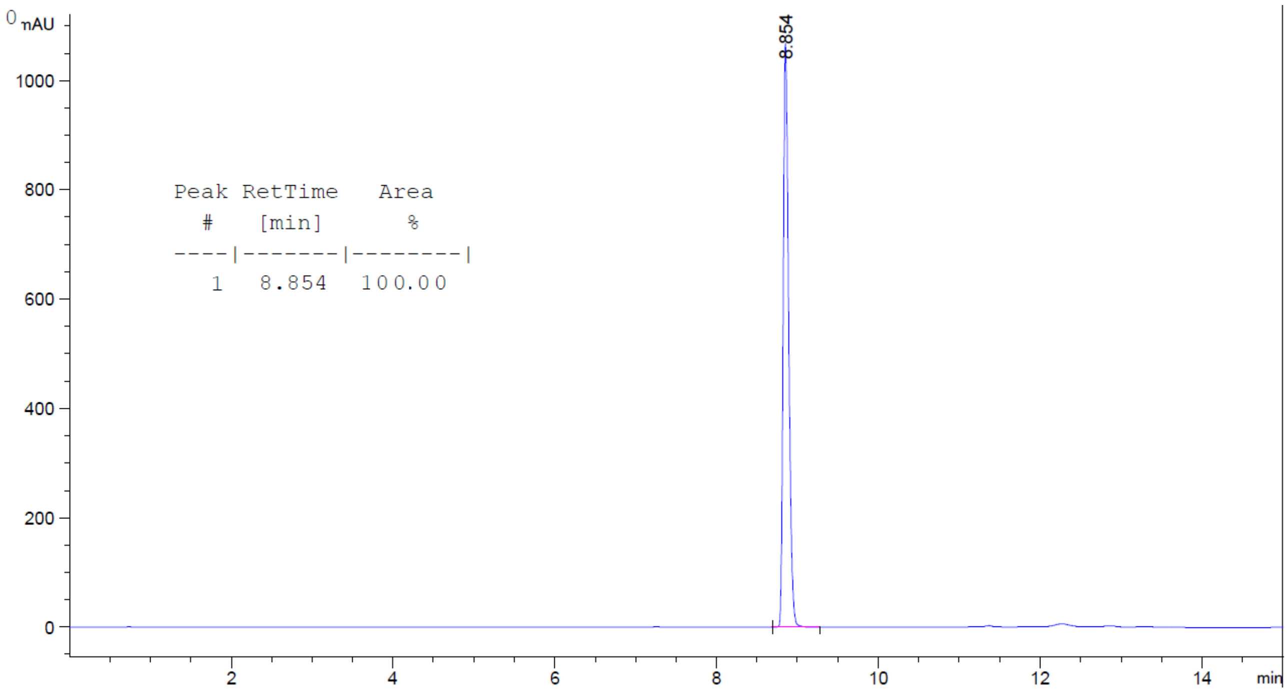

Peak #	RetTime [min]	Area %
1	8.854	100.00

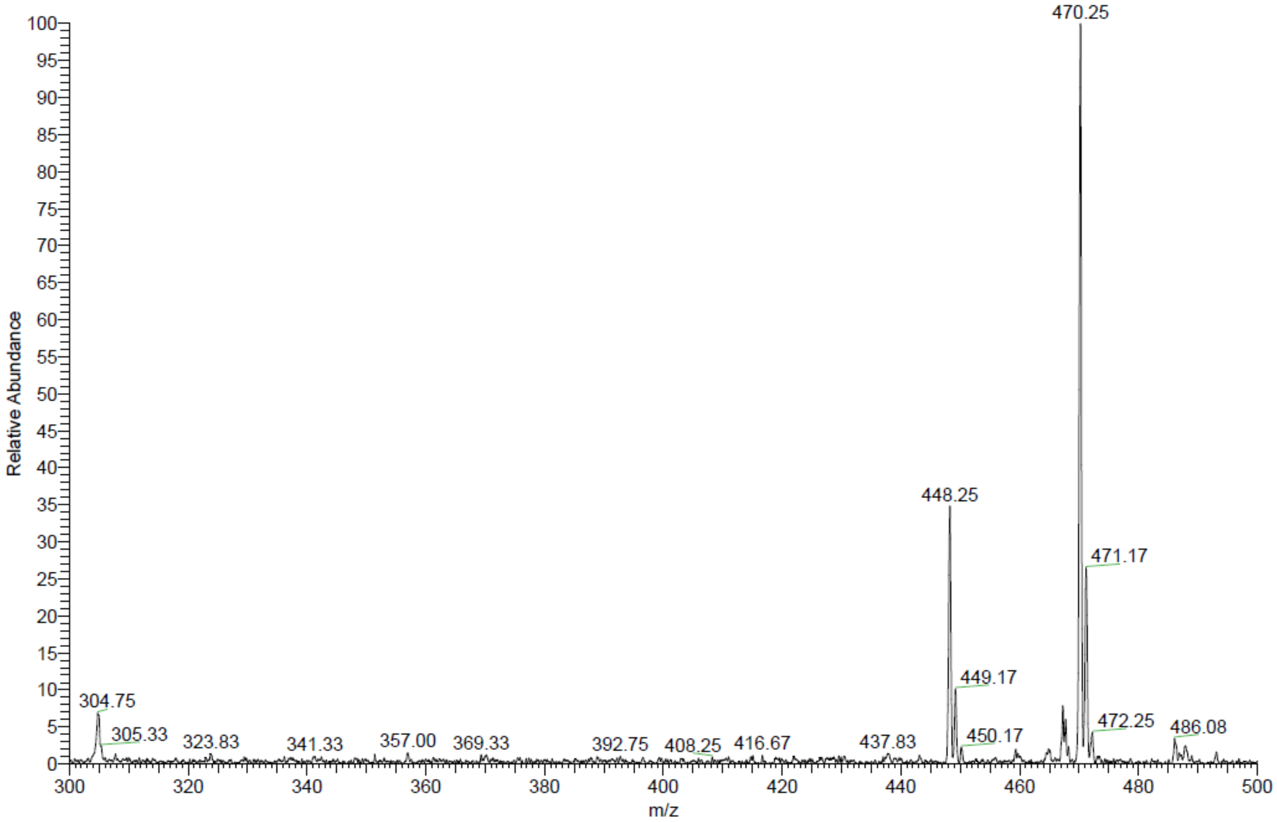

Compound **4**

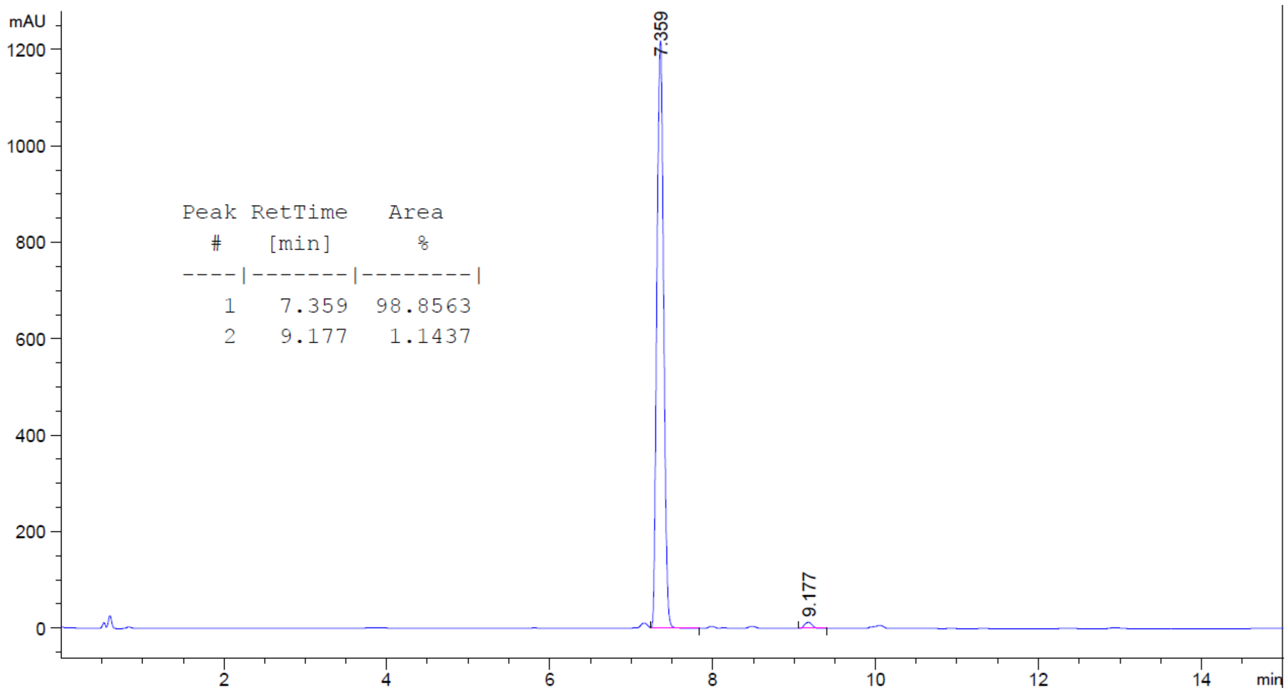
mAU
1200
1000
800
600
400
200
0
7.359
9.177
Peak RetTime Area
[min] %
1 7.359 98.8563
2 9.177 1.1437
2
4
6
8
10
12
14
min

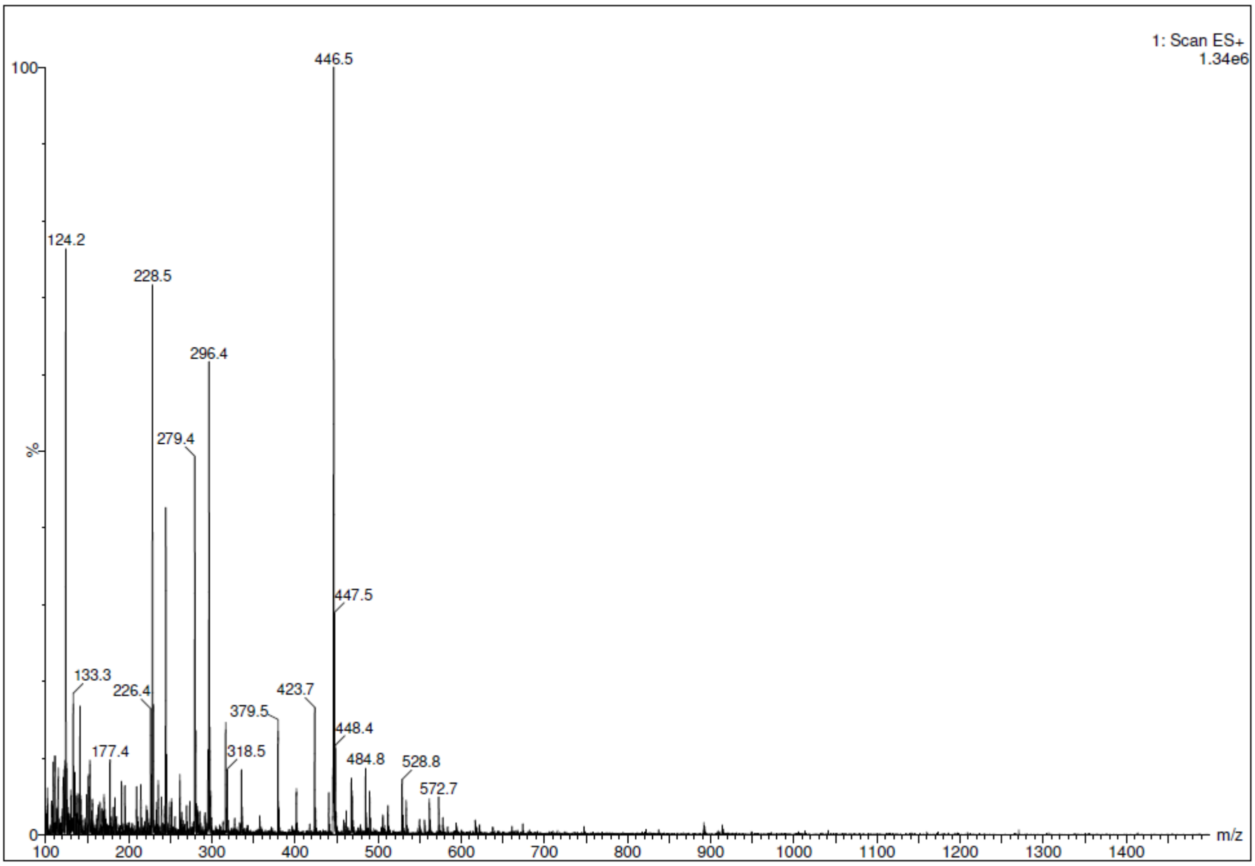
1: Scan ES+
1.34e6
100
%
0
446.5
124.2
228.5
296.4
279.4
447.5
133.3
226.4
177.4
423.7
379.5
318.5
448.4
484.8
528.8
572.7
m/z
100
200
300
400
500
600
700
800
900
1000
1100
1200
1300
1400

Compound **5**

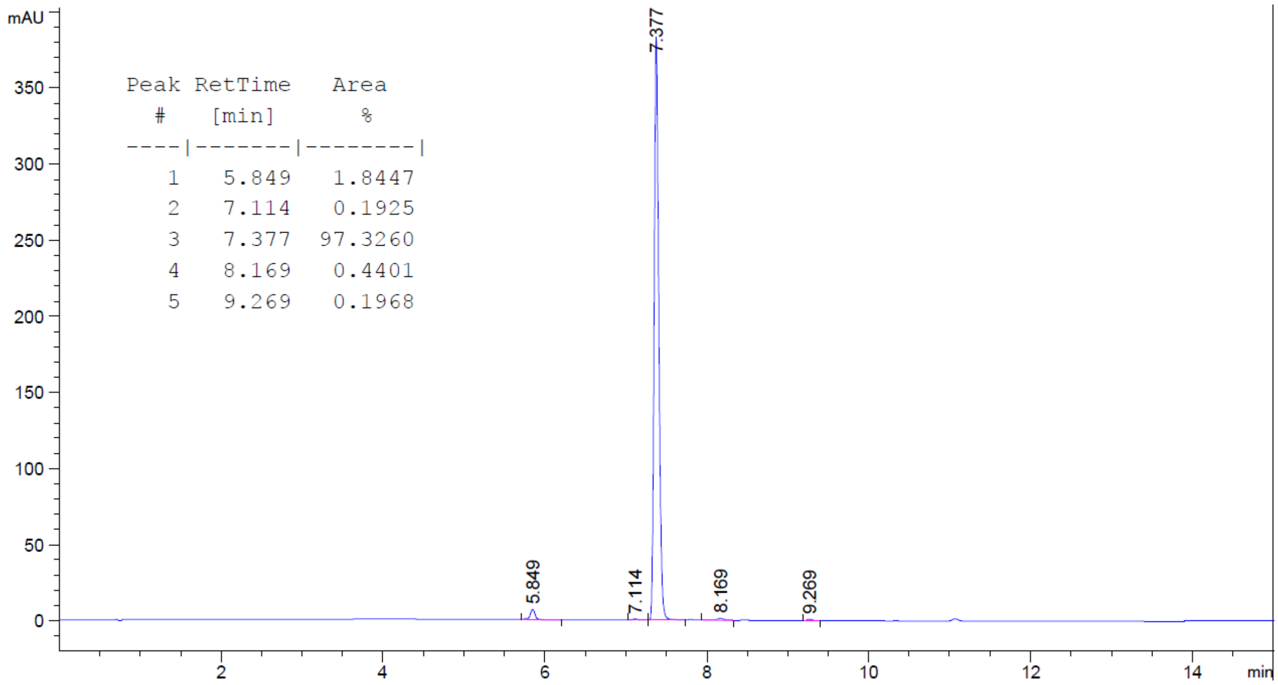

mAU
Peak # | RetTime [min] | Area %
1 5.849 1.8447
2 7.114 0.1925
3 7.377 97.3260
4 8.169 0.4401
5 9.269 0.1968
7.377
5.849
7.114
8.169
9.269
min

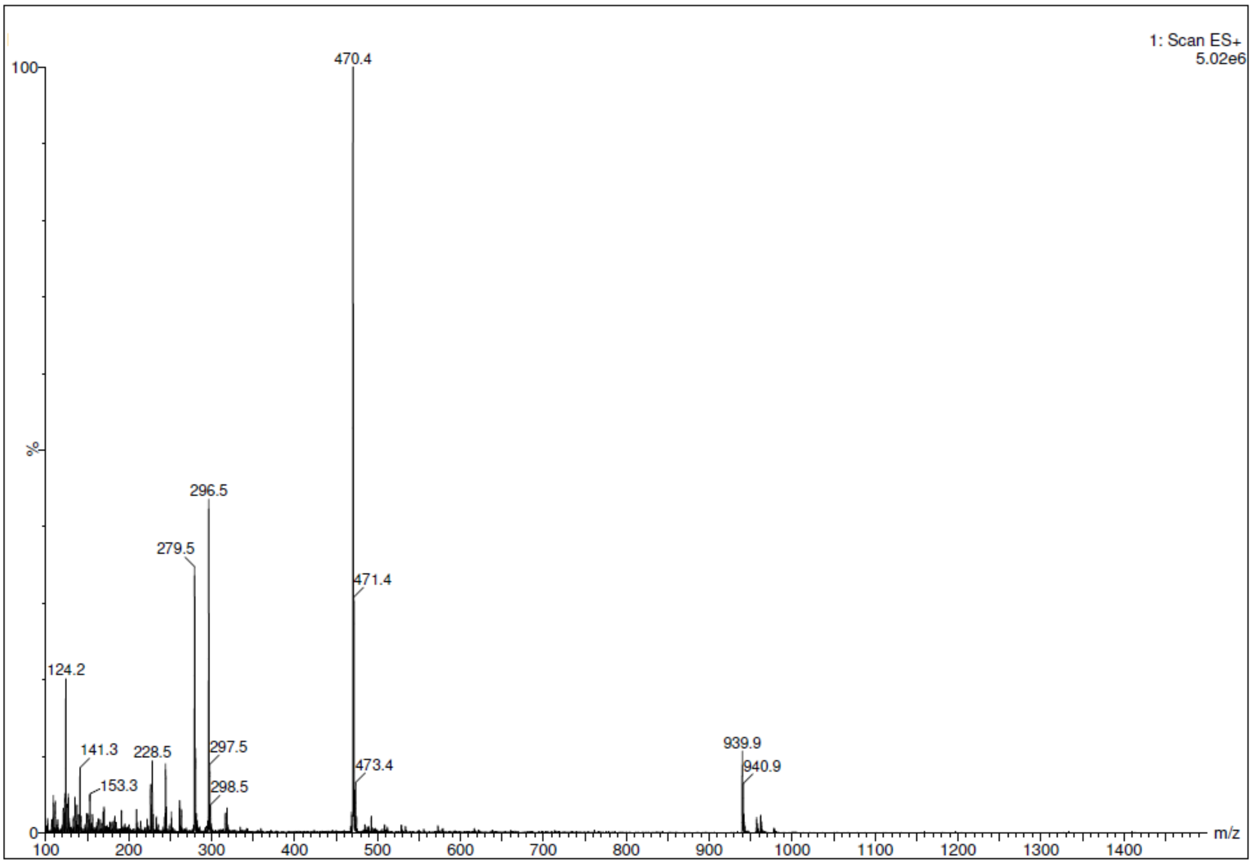

1: Scan ES+
5.02e6
470.4
296.5
279.5
471.4
124.2
141.3
228.5
153.3
297.5
298.5
473.4
939.9
940.9
%
m/z

Compound **6**

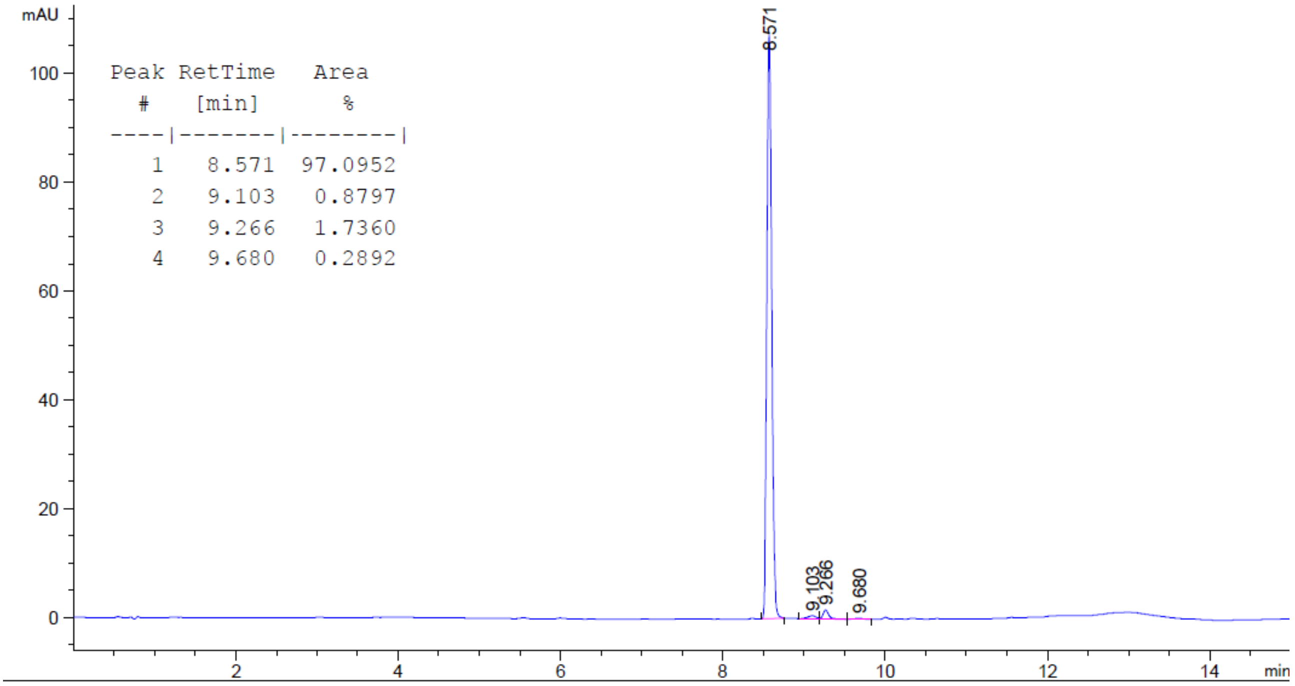
mAU
Peak RetTime Area
[min] %
1 8.571 97.0952
2 9.103 0.8797
3 9.266 1.7360
4 9.680 0.2892
8.571
9.103
9.266
9.680
min

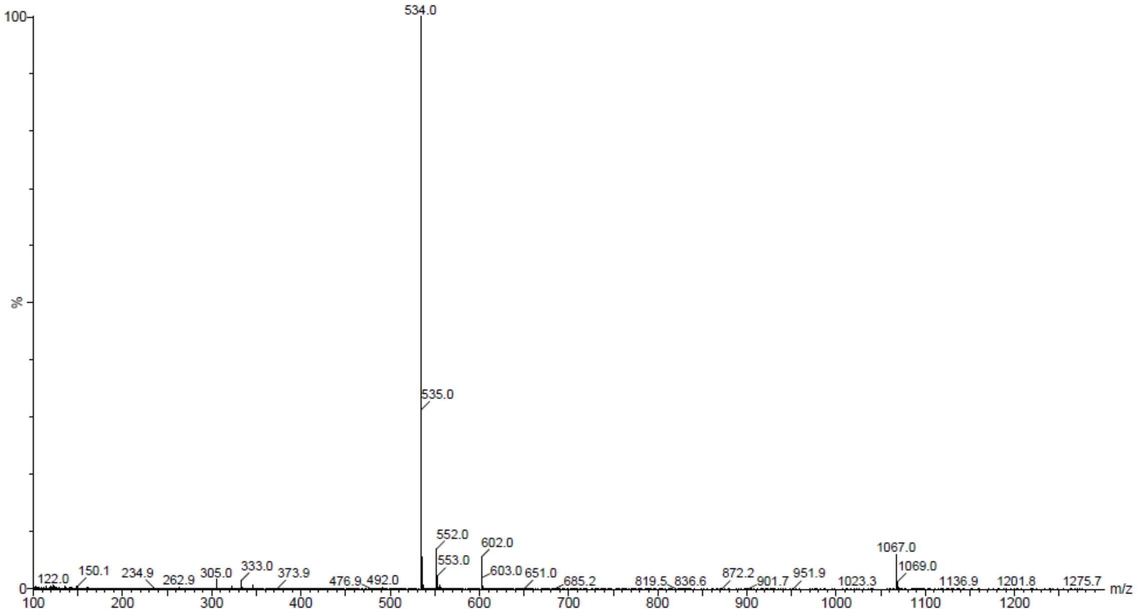
534.0
535.0
552.0
553.0
602.0
603.0
651.0
1067.0
1069.0
%
m/z

Compound **7**

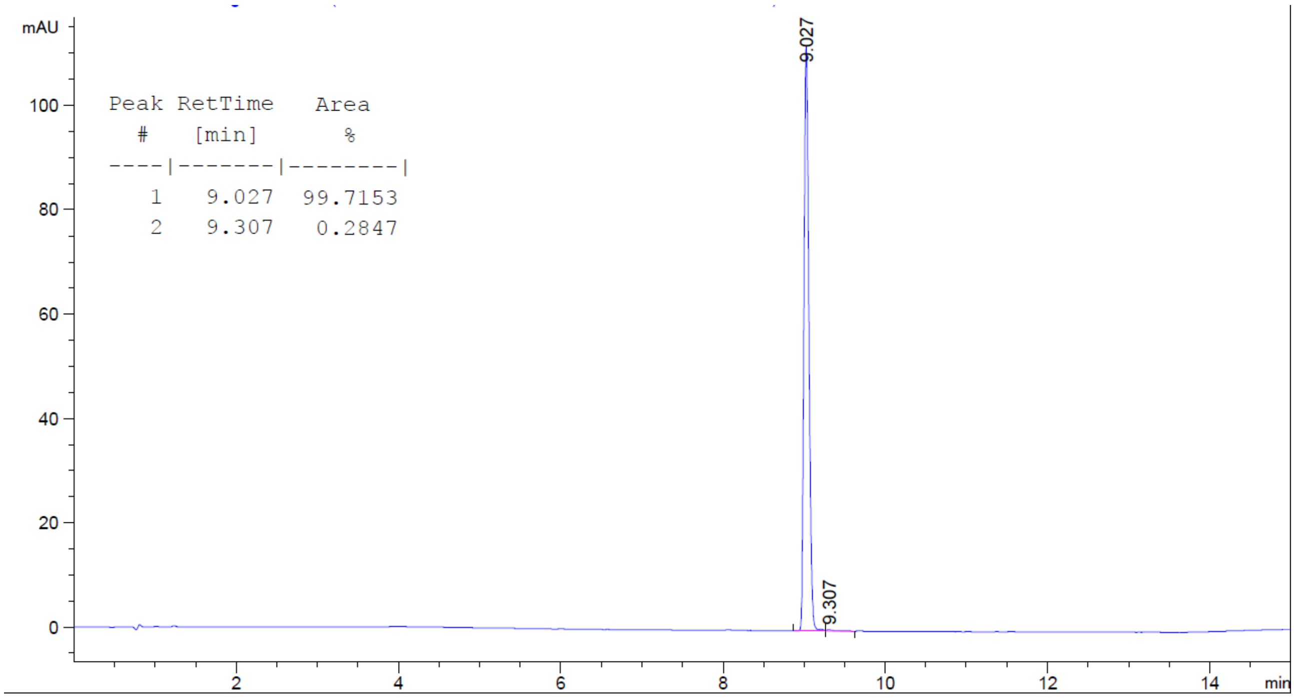

mAU
Peak # | RetTime [min] | Area %
1 9.027 99.7153
2 9.307 0.2847
9.027
9.307
min

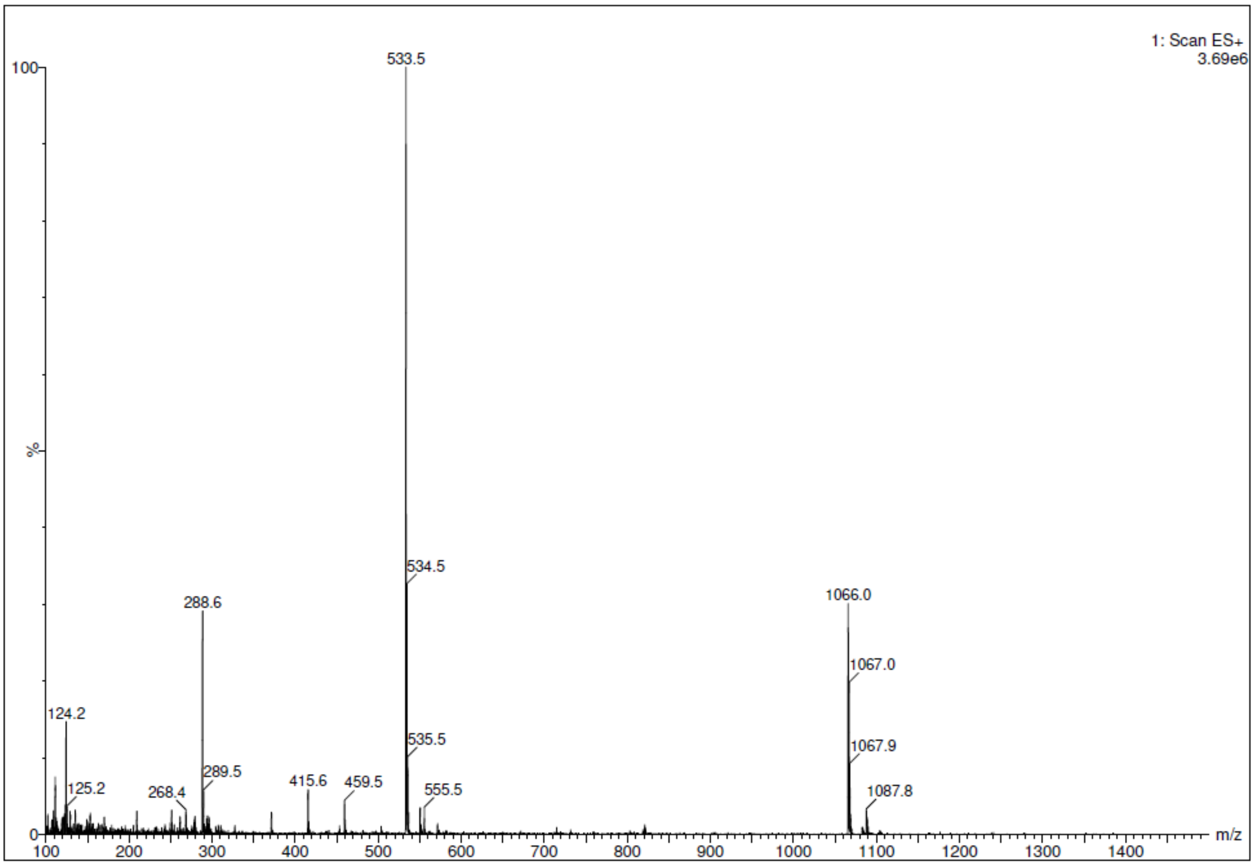

1: Scan ES+
3.69e6
533.5
534.5
288.6
1066.0
1067.0
124.2
535.5
1067.9
289.5
125.2
268.4
415.6
459.5
555.5
1087.8
%
m/z

Compound **15**

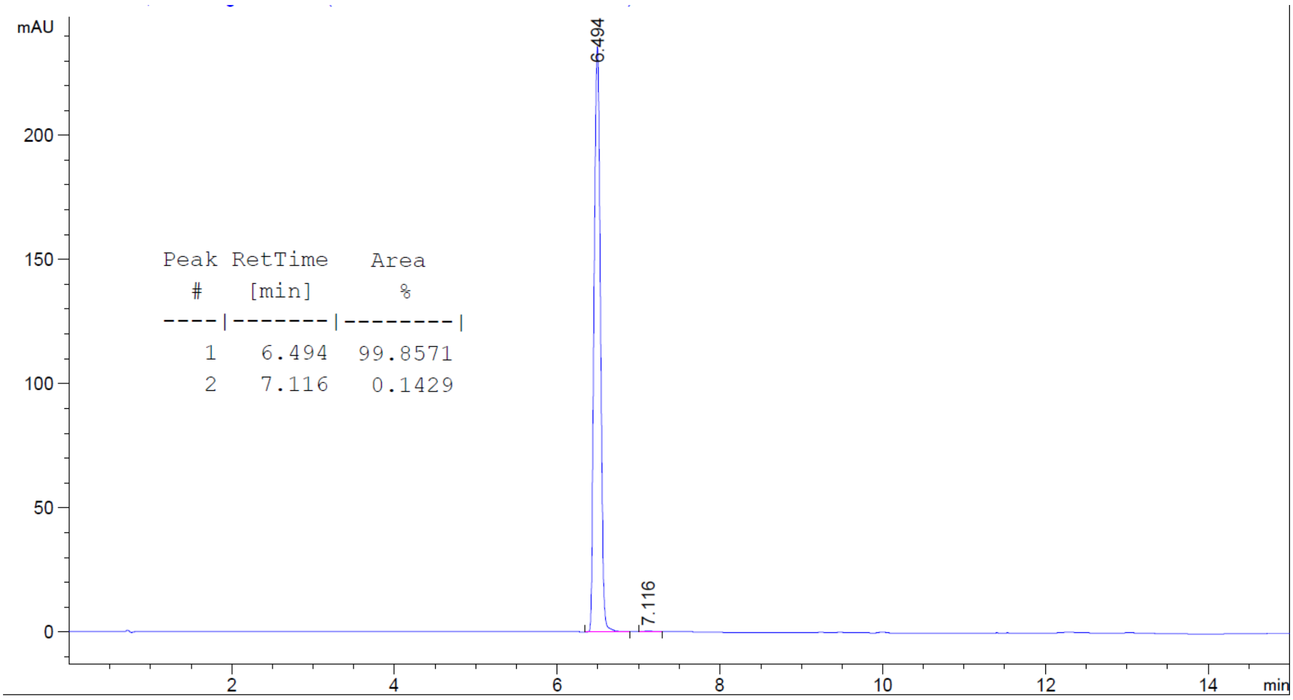

mAU
6.494
7.116
Peak RetTime Area
[min] %
1 6.494 99.8571
2 7.116 0.1429
min

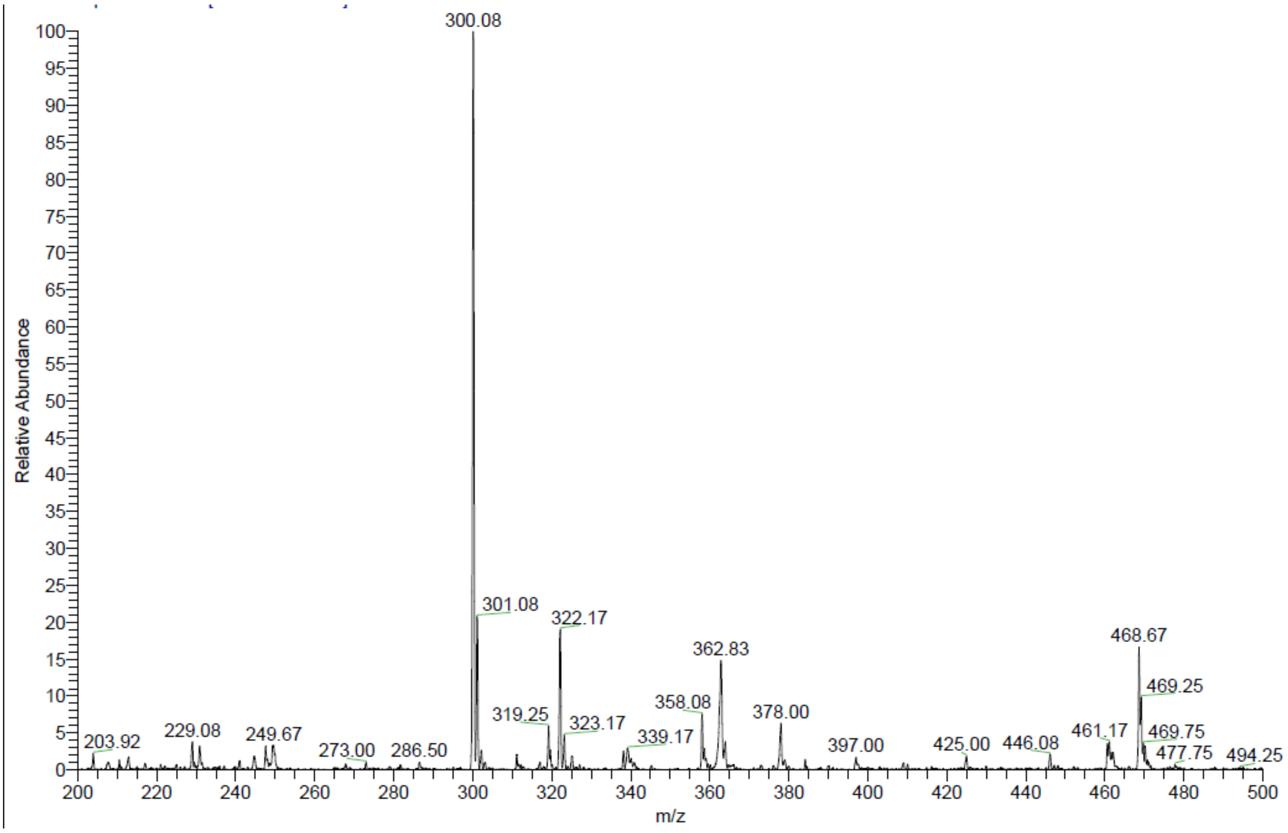

300.08
301.08
322.17
362.83
468.67
469.25
358.08
378.00
319.25
323.17
339.17
461.17
469.75
477.75
494.25
203.92
229.08
249.67
273.00
286.50
397.00
425.00
446.08
Relative Abundance
m/z

Compound **16**

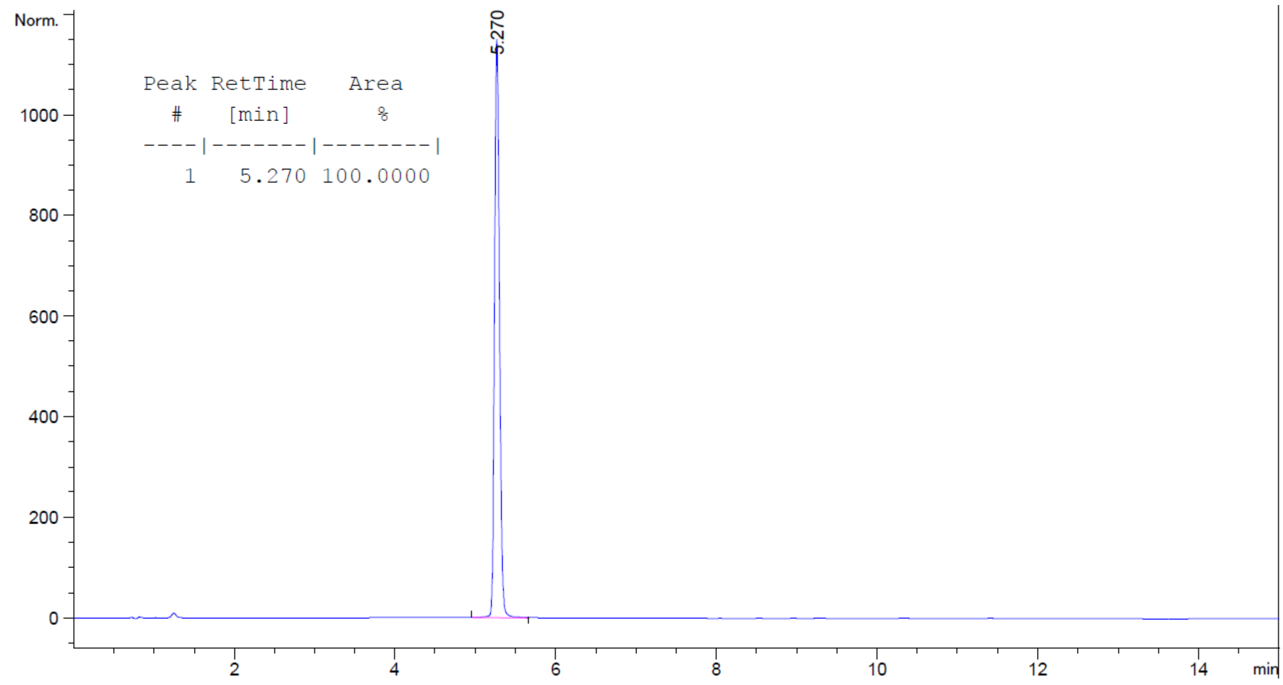
Norm.
5.270
Peak RetTime Area
[min] %
1 5.270 100.0000
min

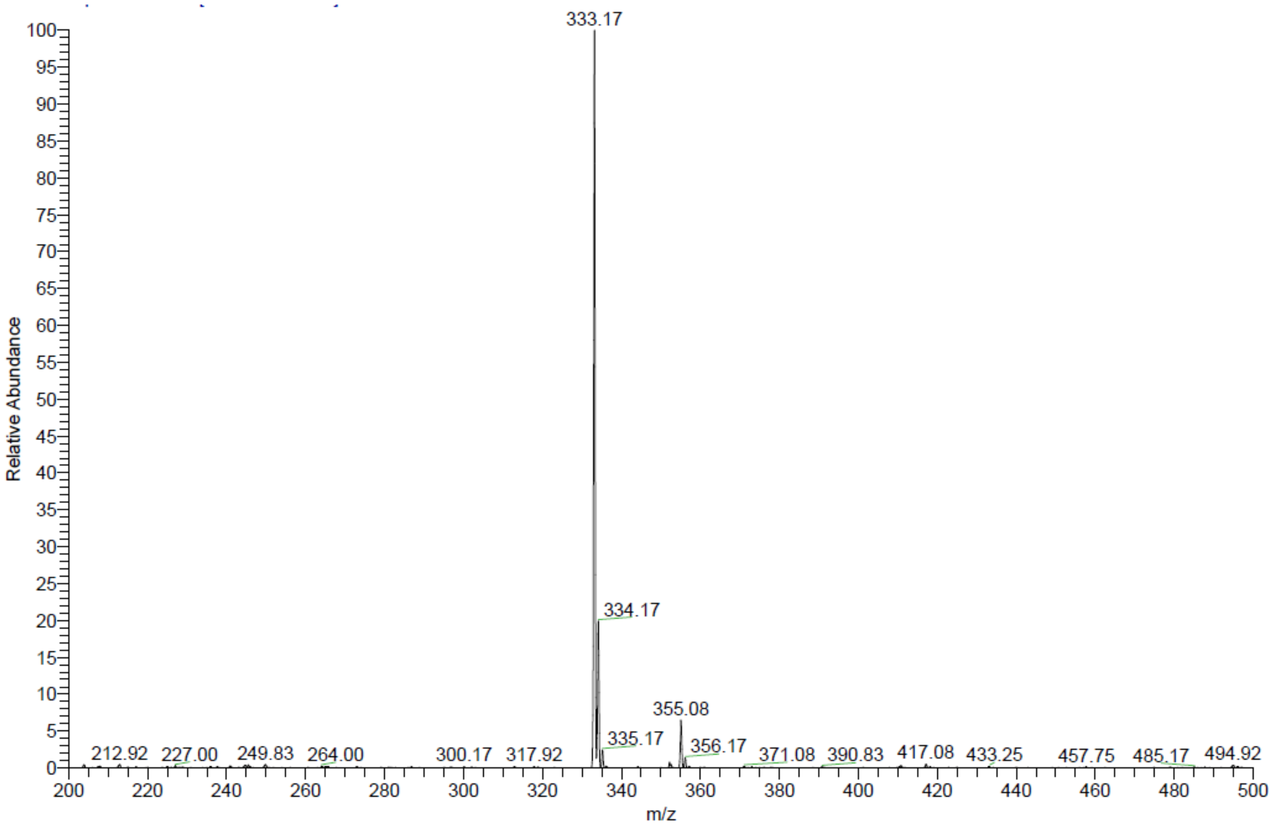
333.17
334.17
355.08
335.17
356.17
Relative Abundance
m/z

NMR characterization:

^{1}H-NMR – $CDCl_3$: **1**

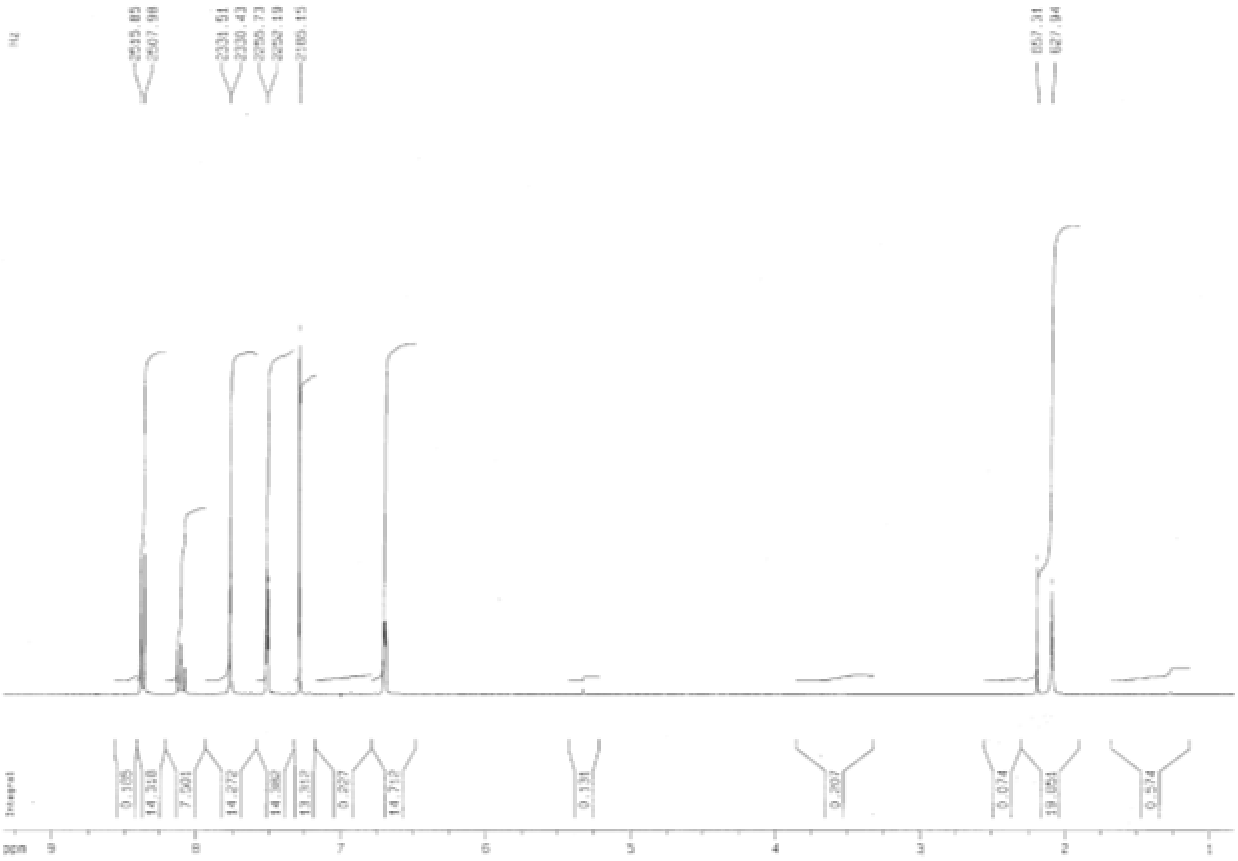

^{13}C-NMR – $CDCl_3$: **1**

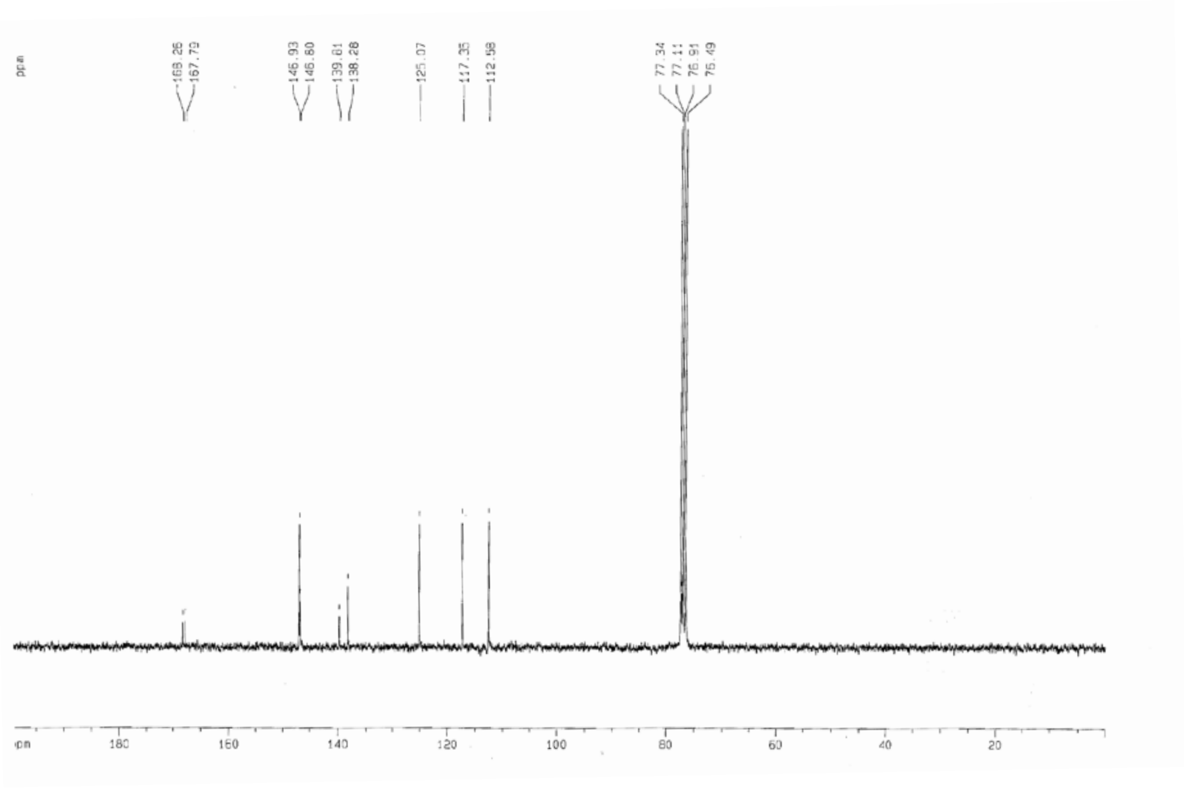

^{1}H-NMR – $CDCl_3$: **2**

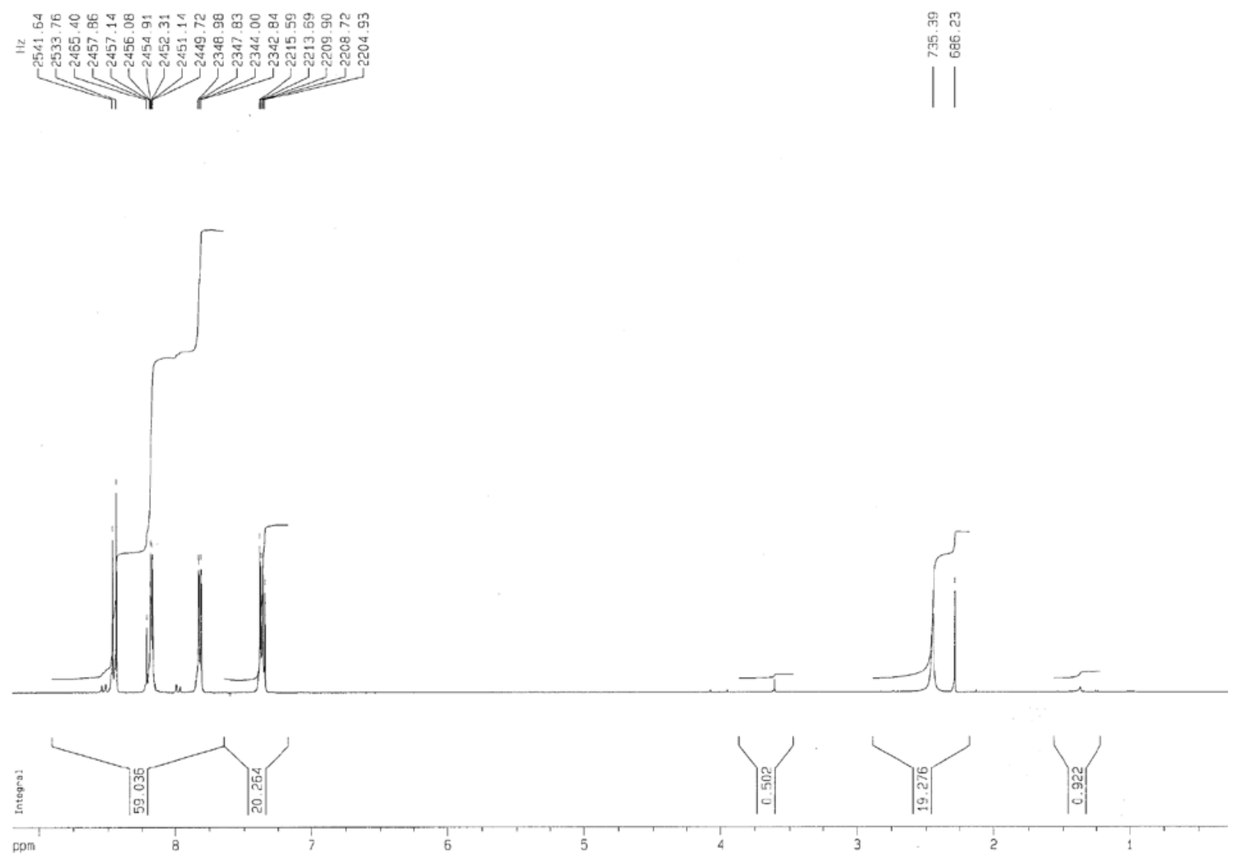

^{13}C-NMR – DMSO d6: **2**

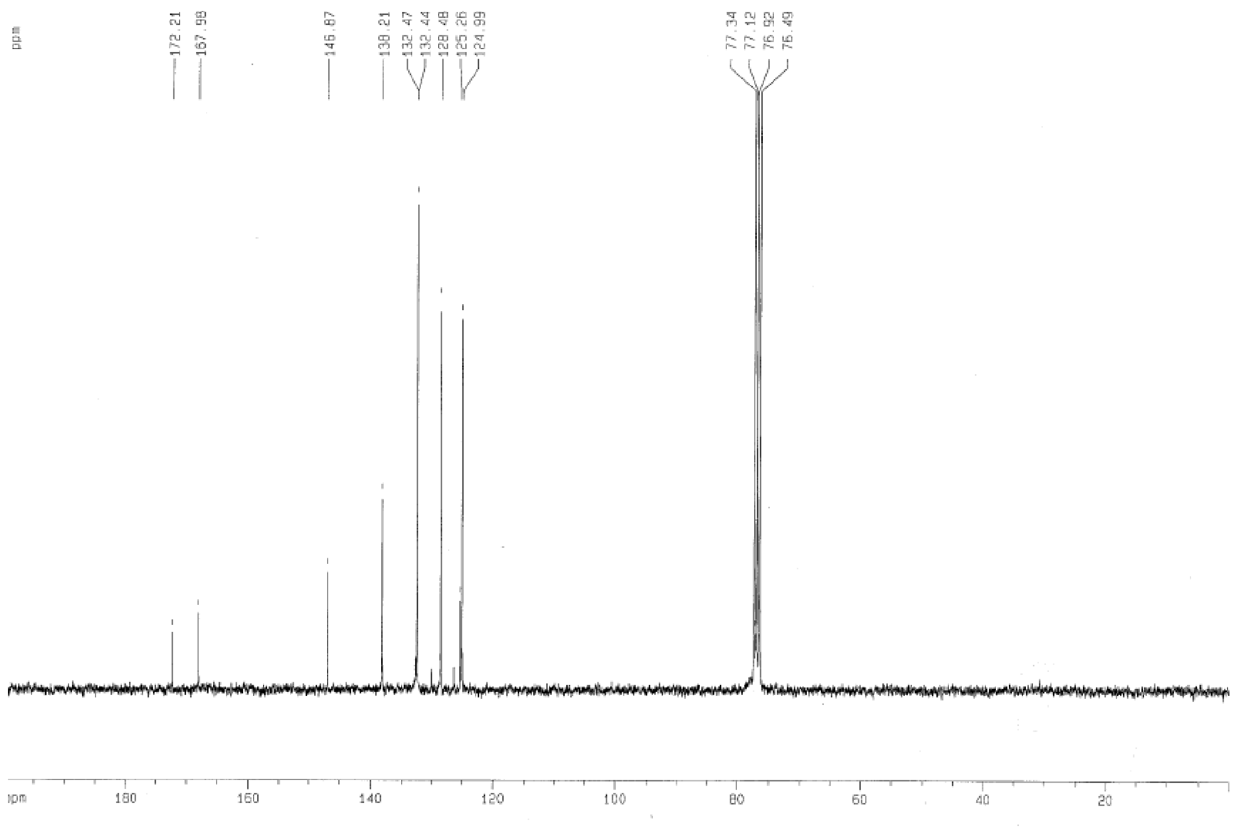

^{1}H-NMR – $CDCl_3$: **3**

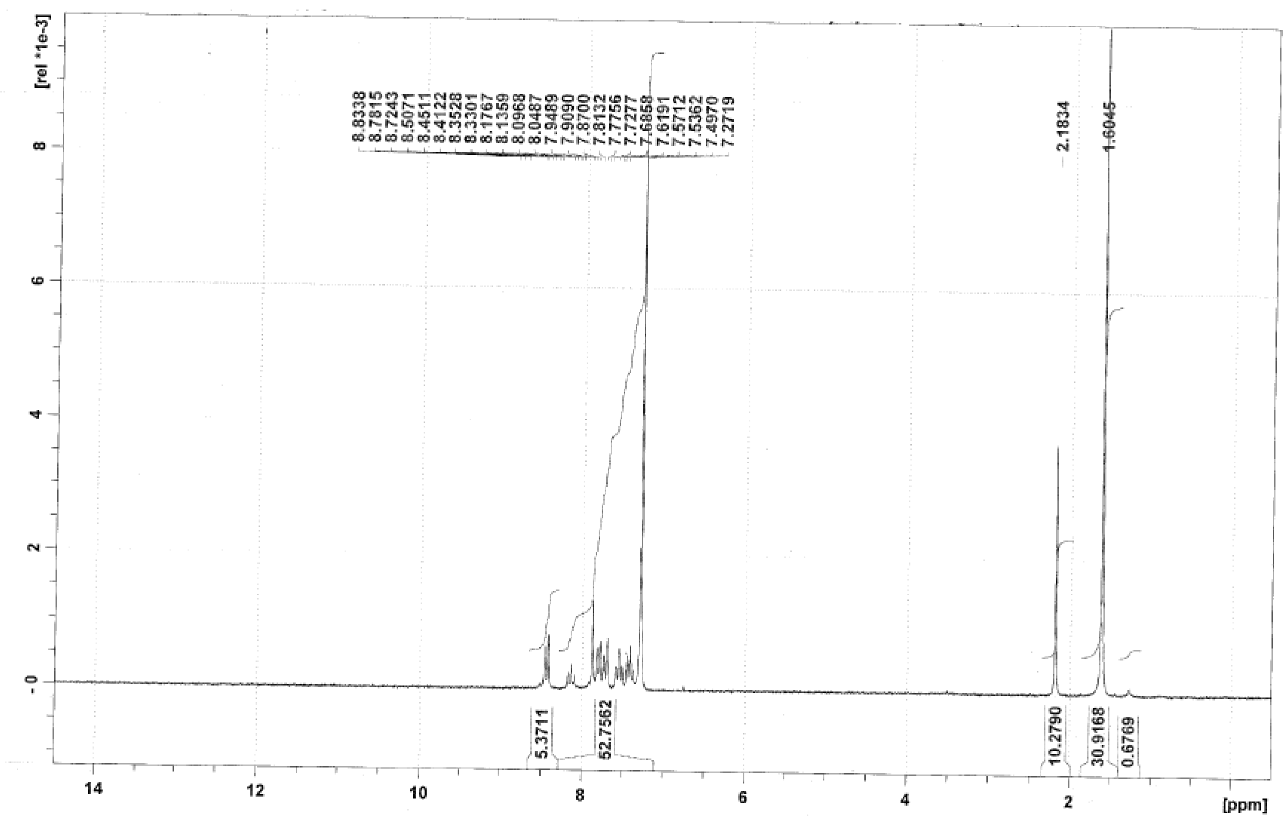

^{13}C-NMR – $CDCl_3$: **3**

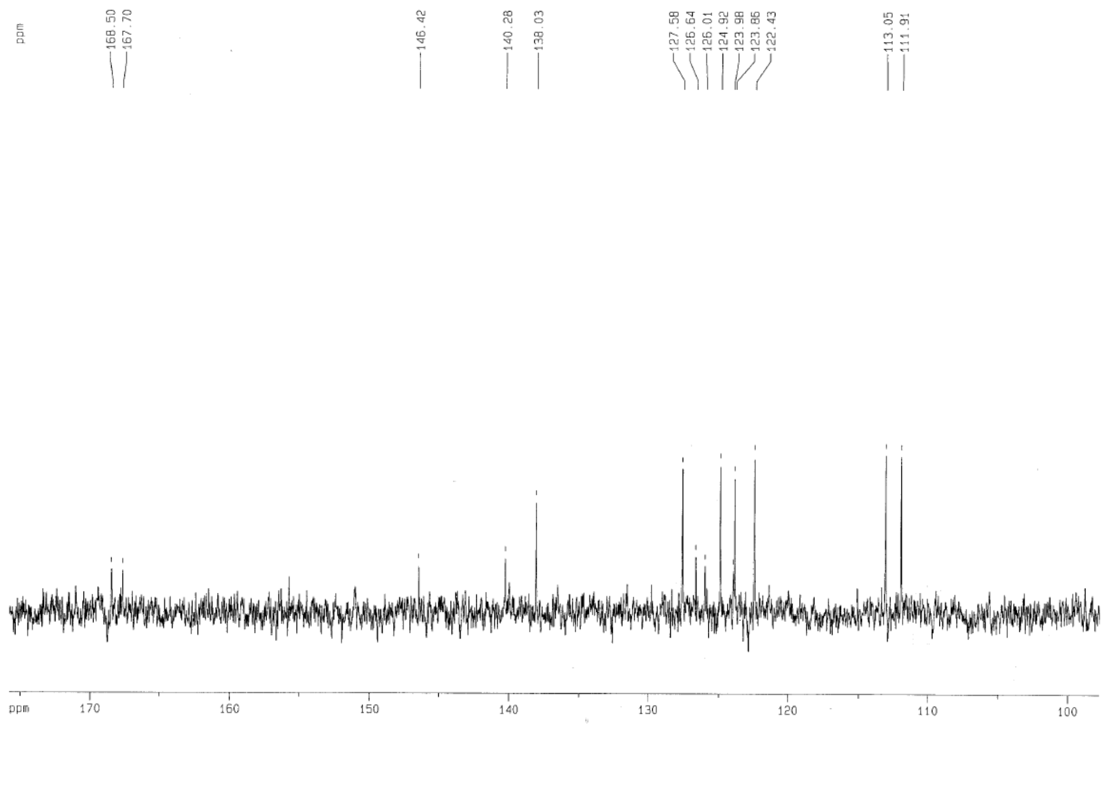

^{1}H-NMR – DMSO d6: **4**

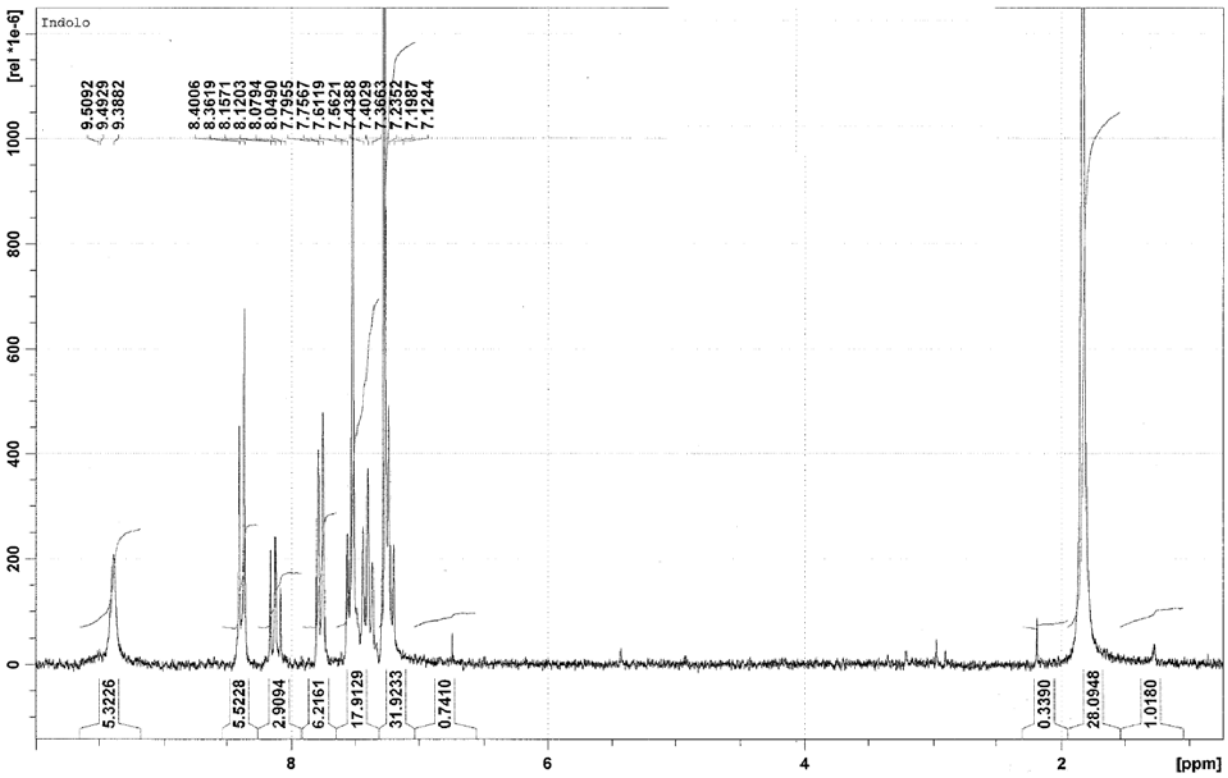

^{1}H-NMR – DMSO d6: Fragment **5**

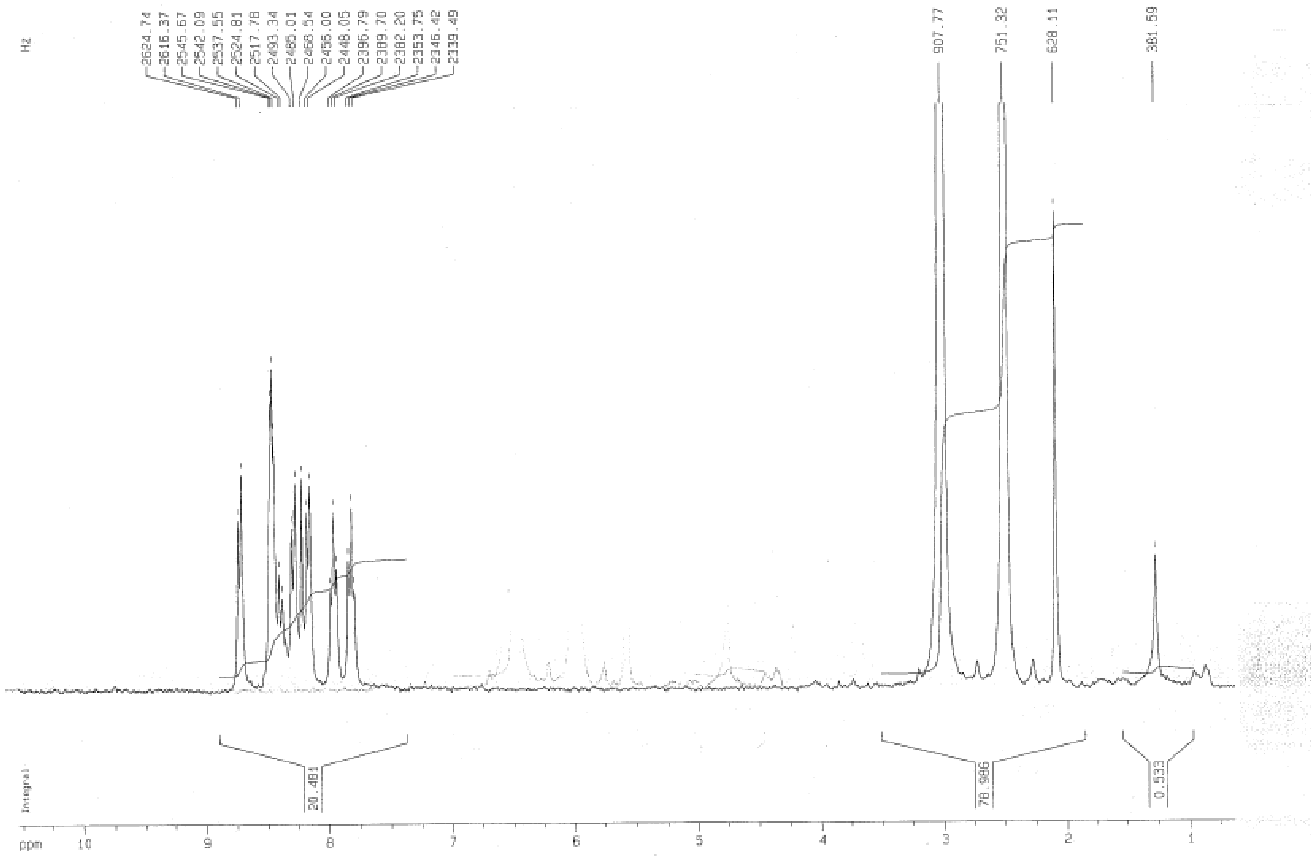

^{1}H-NMR – DMSO d6: Fragment **6**

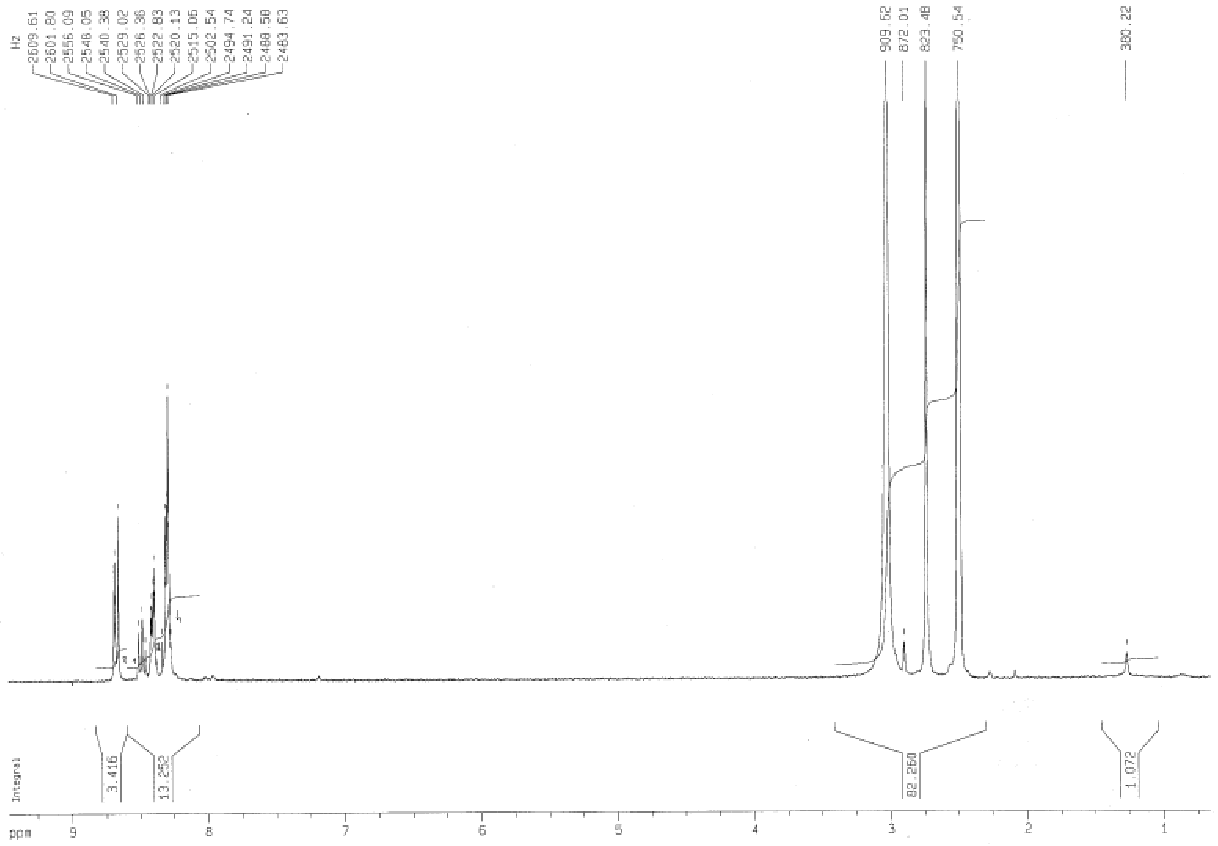

^{1}H-NMR – $CDCl_3$: Fragment **7**

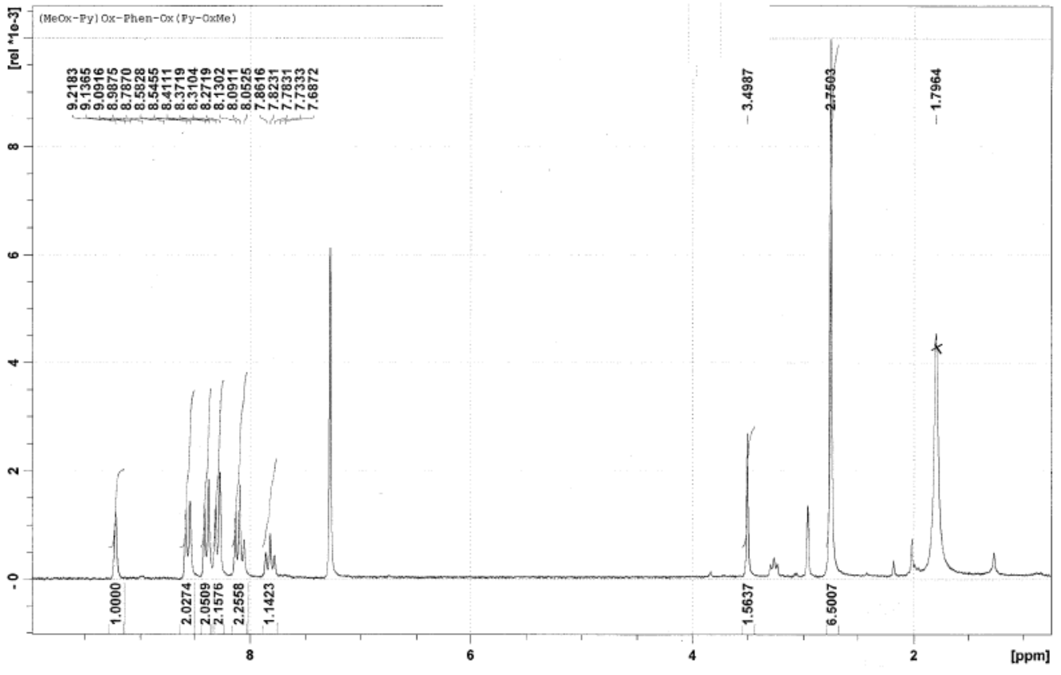

^{1}H-NMR – DMSO d6: **15**

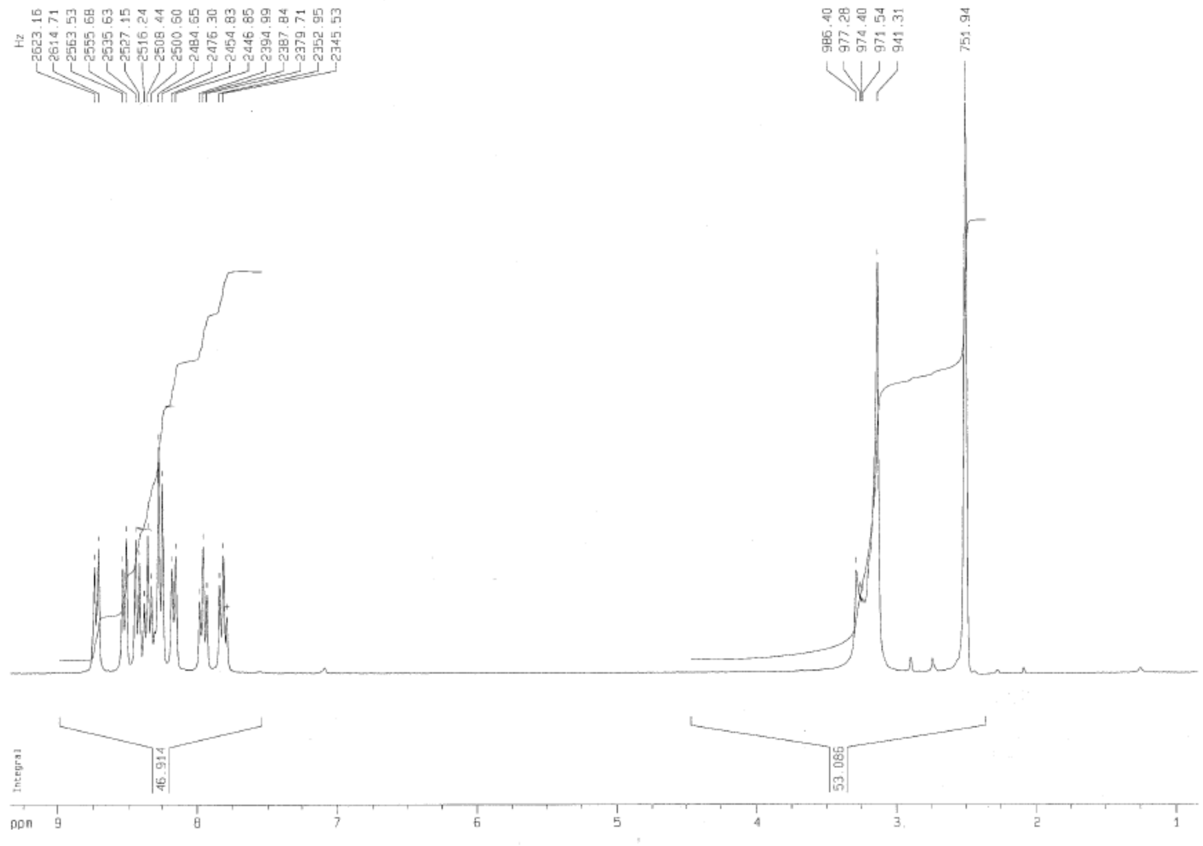

^{13}C-NMR – DMSO d6: **15**

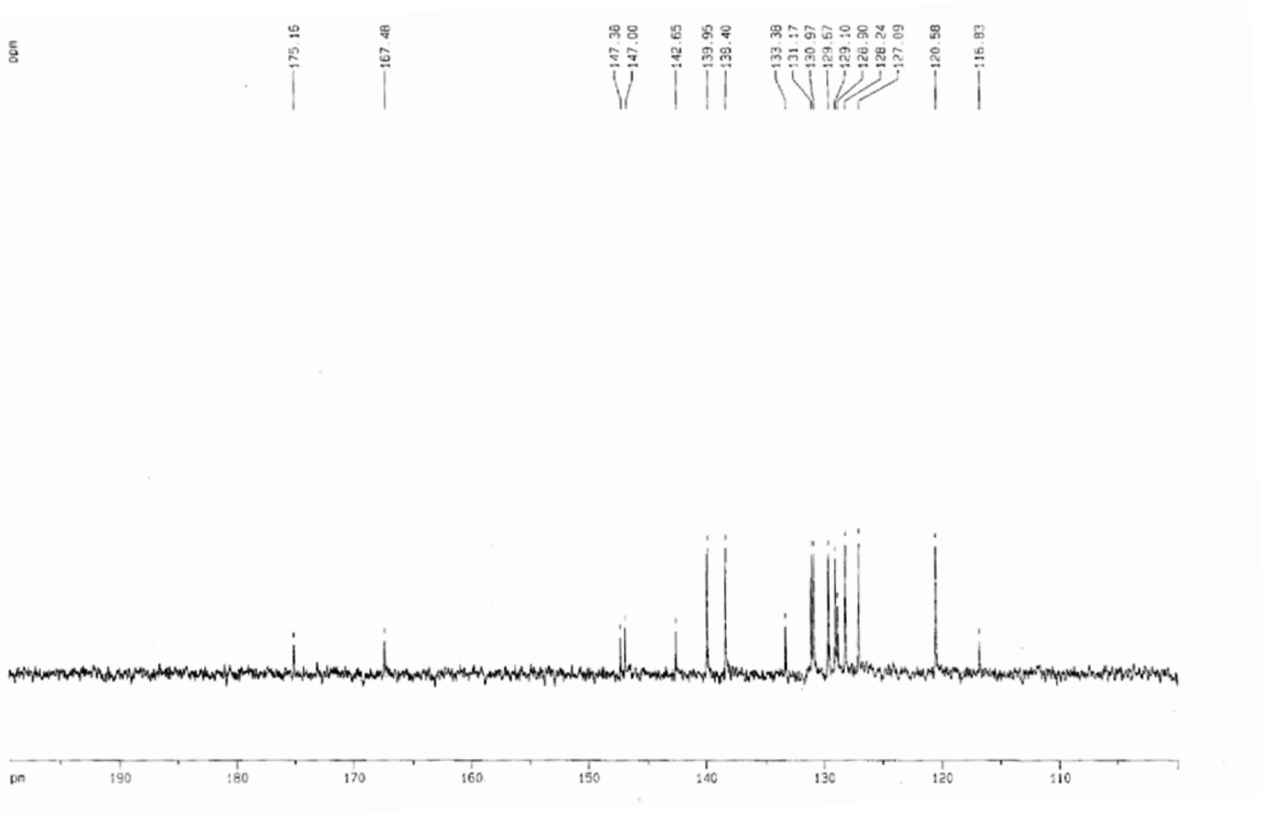

^{1}H-NMR – DMSO d6: **16**

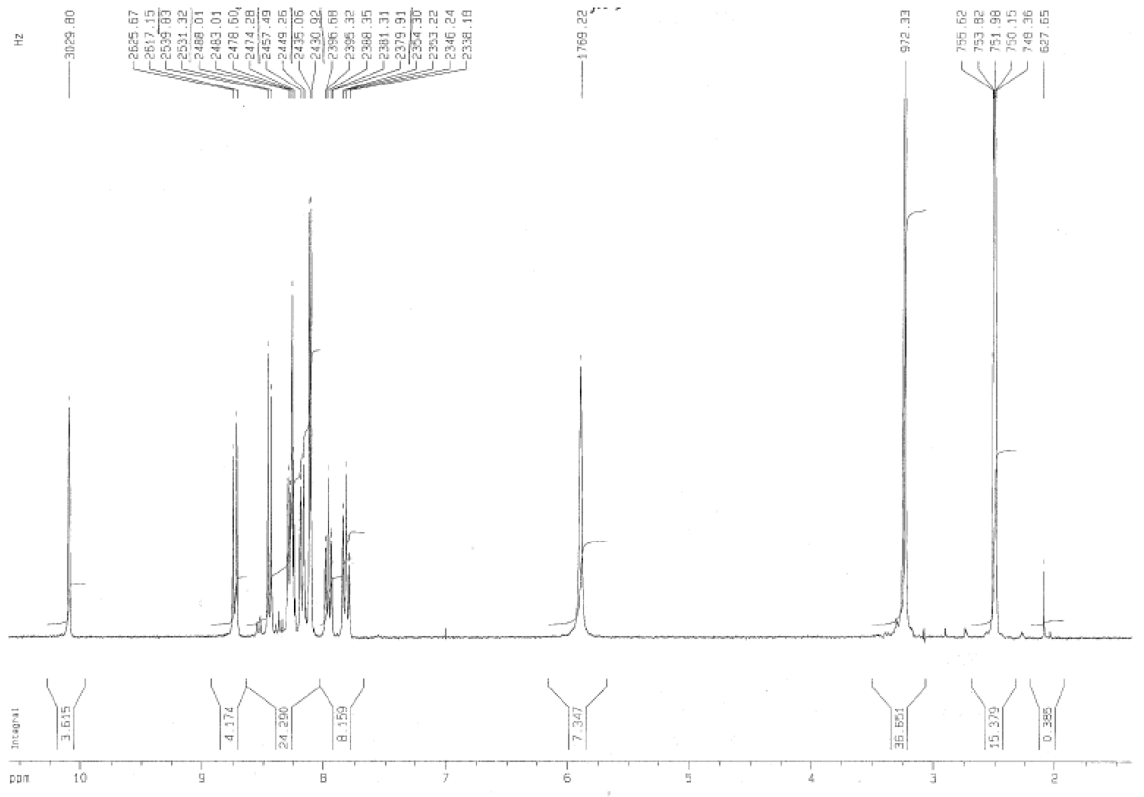

^{13}C-NMR – DMSO d6: **16**

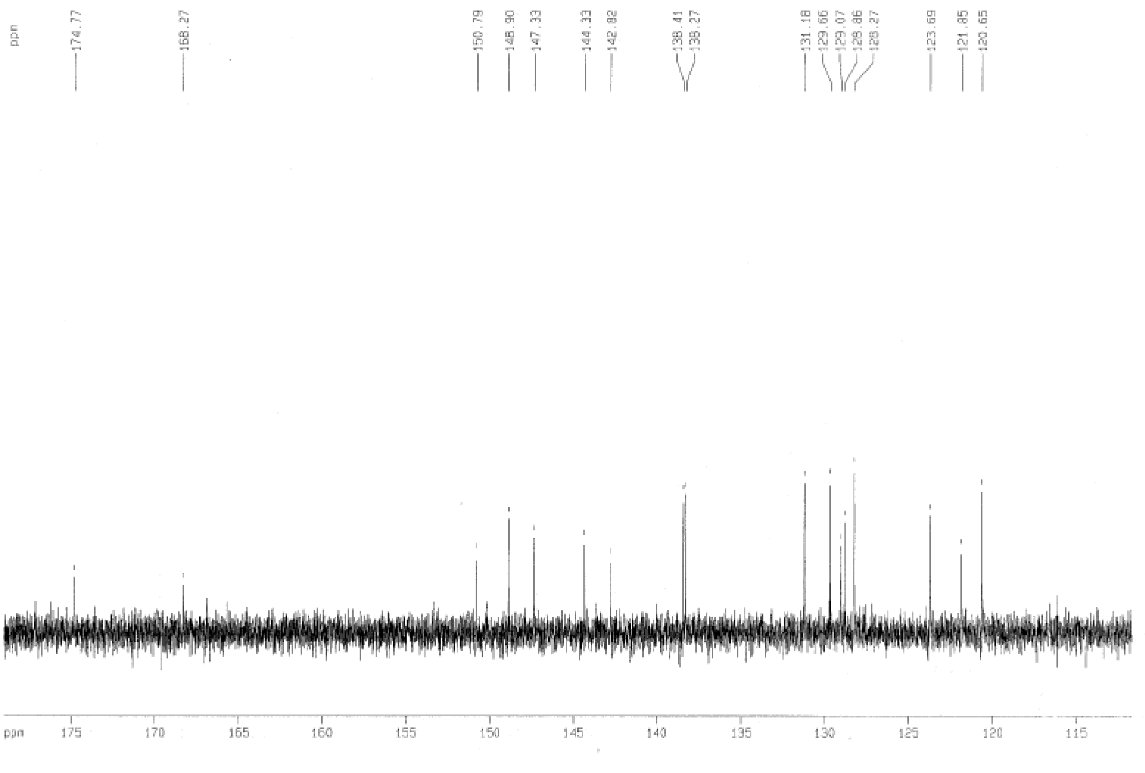

^{1}H-NMR – $CDCl_3$: **17**

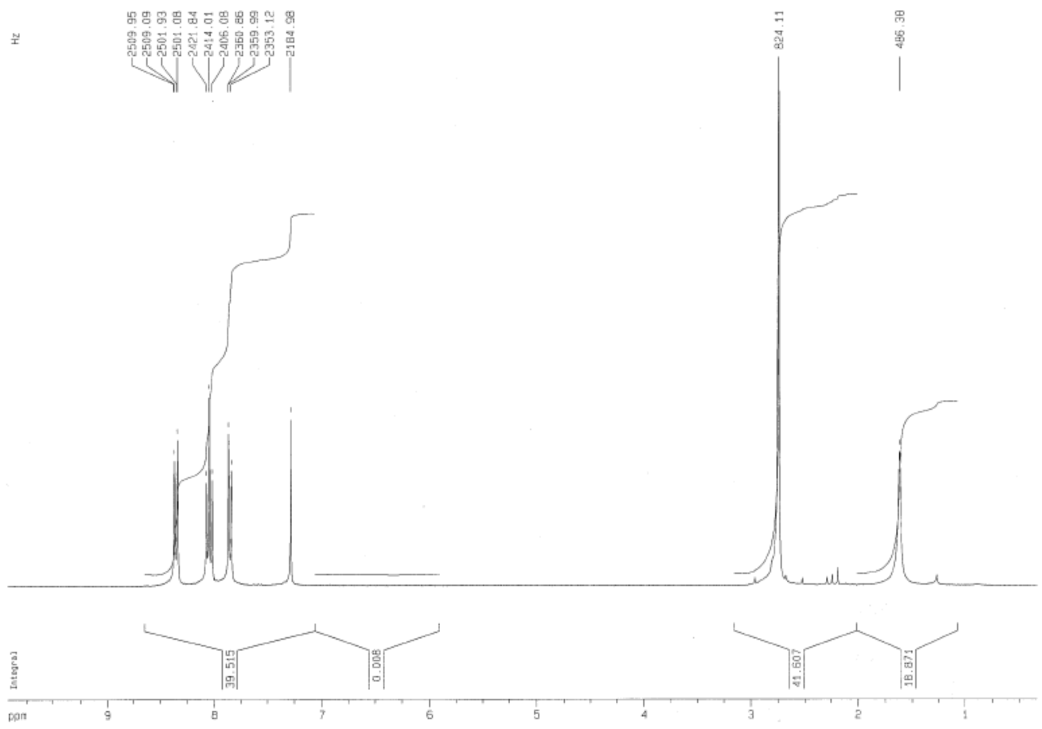

^{13}C-NMR – $CDCl_3$ d6: **17**

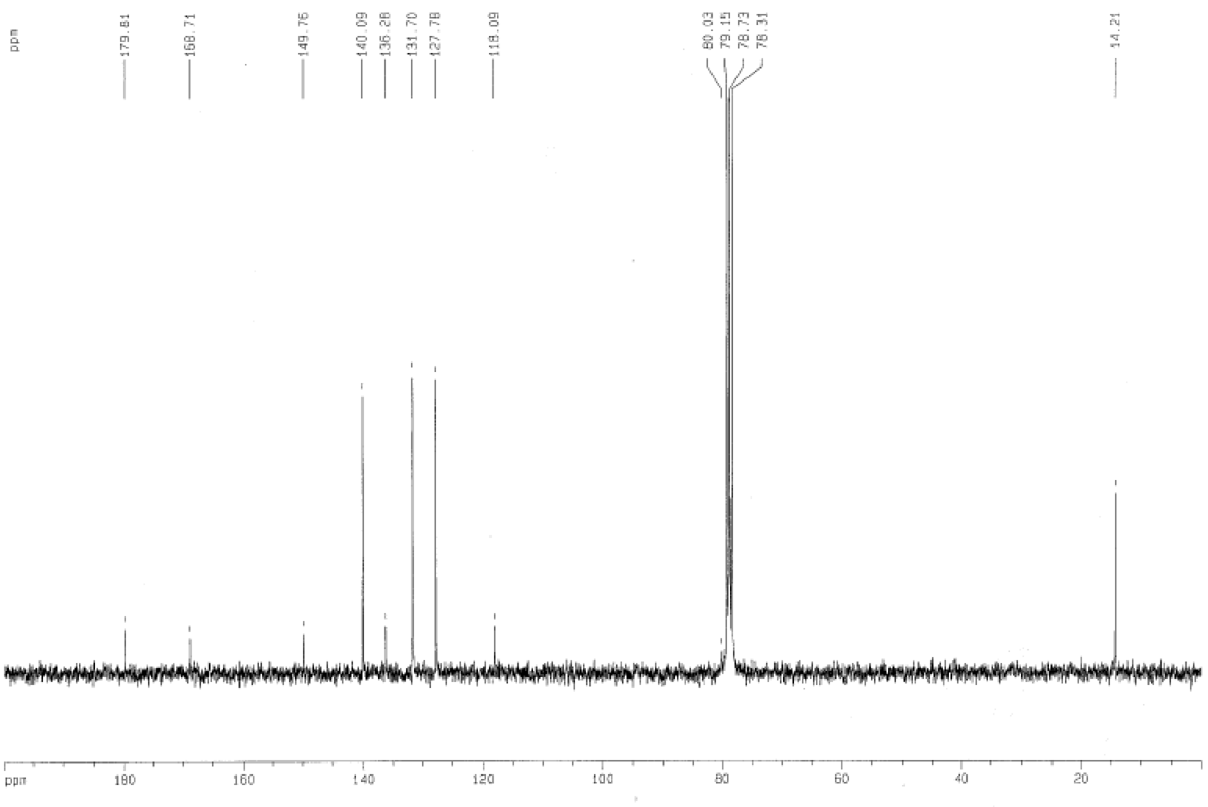

^{1}H-NMR – DMSO d6: **18**

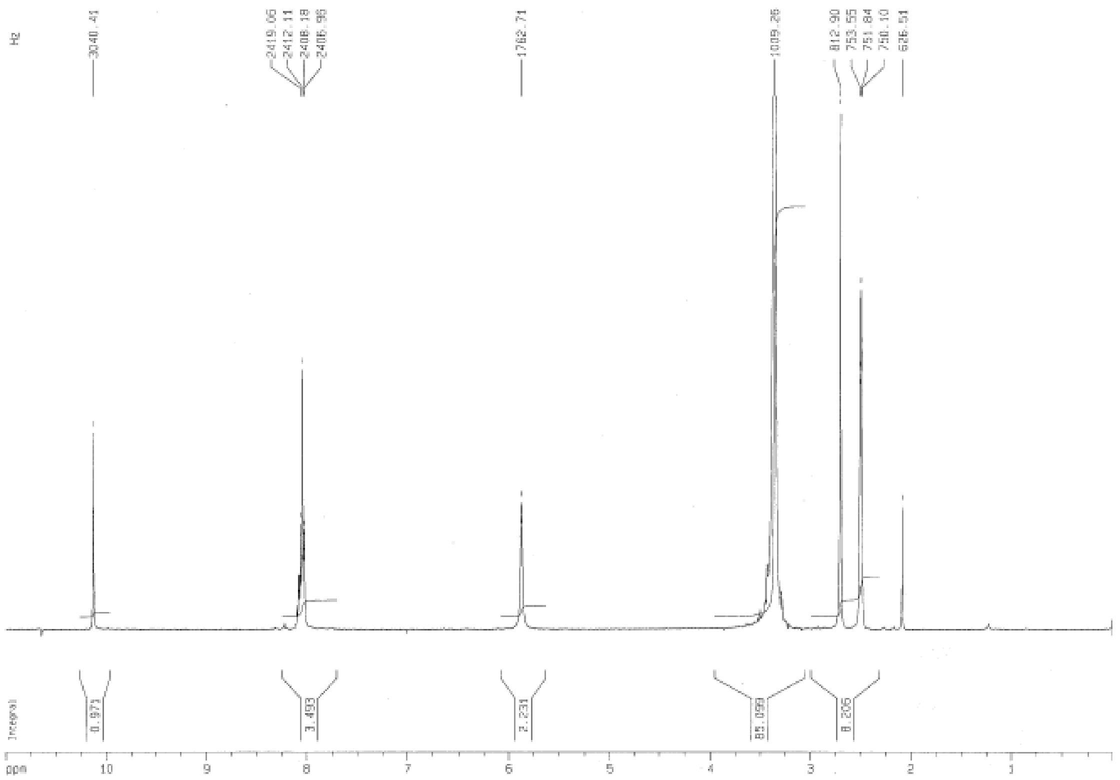

^{13}C-NMR – DMSO d6: **18**

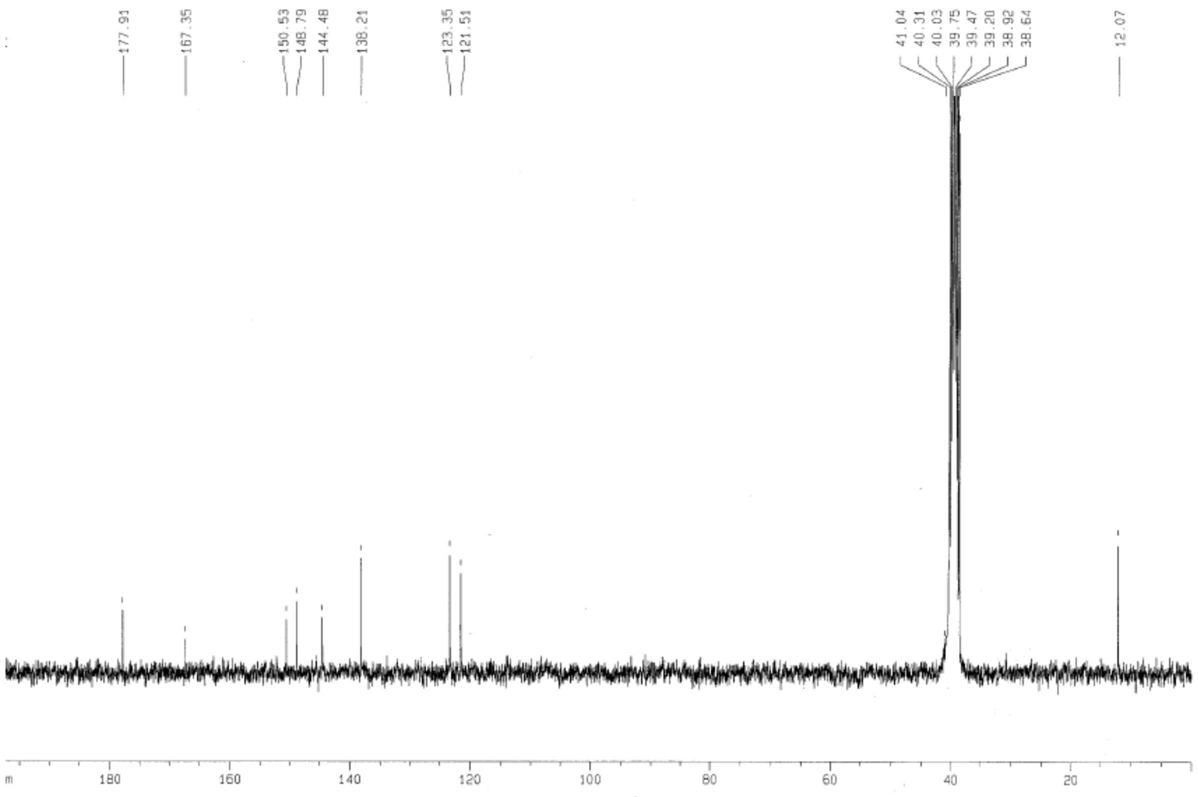

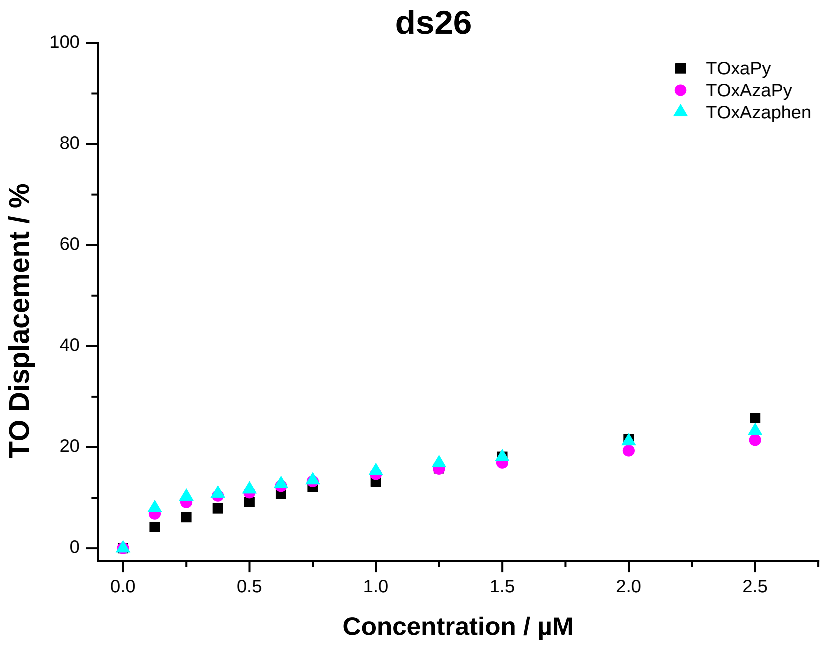

Figure S1: G4-FID plots of TOxAzaPy and TOxAzaPhen in the presence of ds26 (ds DNA). TOxaPy is used as a reference compound.

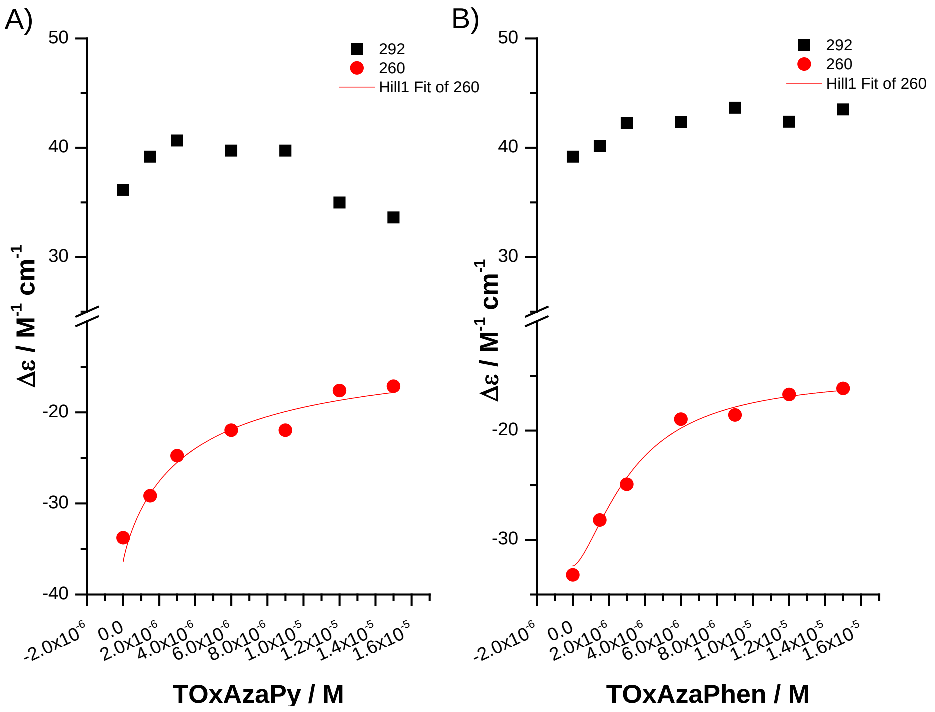

Figure S2: Titration plots representing the change in ellipticity of 22AG in Na$^+$-rich buffer at 292 nm (black squares) and 260 nm (red dots), with the addition of TOxAzaPy A) and TOxAzaPhen B). Variation of the ellipticity as a function of concentration of ligand A) TOxAzaPyand B) TOxAzaPhen. Lines in A) and B) correspond to the non-linear regressions performed as fitting at 260 nm.
